# Rumination and Rebound from Failure as a Function of Gender and Time on Task

**DOI:** 10.3390/brainsci6010007

**Published:** 2016-02-17

**Authors:** Ronald C. Whiteman, Jennifer A. Mangels

**Affiliations:** 1 Department of Psychology, The Graduate Center at the City University of New York (CUNY), New York, NY 10016, USA; jennifer.mangels@baruch.cuny.edu; 2 Department of Psychology, Baruch College at the City University of New York (CUNY), New York, NY 10010, USA

**Keywords:** brooding, reflection, ERP, feedback-related negativity, FRN, late positive potential, LPP, attention, emotion

## Abstract

Rumination is a trait response to blocked goals that can have positive or negative outcomes for goal resolution depending on where attention is focused. Whereas “moody brooding” on affective states may be maladaptive, especially for females, “reflective pondering” on concrete strategies for problem solving may be more adaptive. In the context of a challenging general knowledge test, we examined how Brooding and Reflection rumination styles predicted students’ subjective and event-related responses (ERPs) to negative feedback, as well as use of this feedback to rebound from failure on a later surprise retest. For females only, Brooding predicted unpleasant feelings after failure as the task progressed. It also predicted enhanced attention to errors through both bottom-up and top-down processes, as indexed by increased early (400–600 ms) and later (600–1000 ms) late positive potentials (LPP), respectively. Reflection, despite increasing females’ initial attention to negative feedback (*i.e.*, early LPP), as well as both genders’ recurring negative thoughts, did not result in sustained top-down attention (*i.e.*, late LPP) or enhanced negative feelings toward errors. Reflection also facilitated rebound from failure in both genders, although Brooding did not hinder it. Implications of these gender and time-related rumination effects for learning in challenging academic situations are discussed.

## 1. Introduction

Failing to achieve our goals can be disappointing at best, and devastating at worst. For some individuals, however, failure elicits a particular flurry of negative thoughts and feelings that come to monopolize their attention at the expense of other goal-relevant information. Given that the ability to learn and rebound from failure is a key predictor of life-long success (e.g., [1,2]), it is important to understand how our cognition and emotions interact to influence goal-directed behaviors such as the ability to overcome failure (e.g., [3,4]). In the present study, we use both behavioral and event-related potential (ERP) measures to examine how rumination, a cognitive response to negative mood states, stressful situations, or adverse life events [5], impacts the response to negative feedback and the ability to effectively learn from it.

Rumination is characterized by repetitive and persistent evaluation of the meaning, causes, and consequences of one’s affective state and personal concerns [6]. Although ruminators often claim that this style of thinking helps them to down-regulate unwanted emotions and arrive at useful insight for solving problems, instead, it often increases negative affect and impairs problem solving [7]. According to recent social-cognitive models of rumination, such as Martin and Tesser’s Control Theory (Control Theory; [8,9]), rumination occurs in the presence of blocked goals, as an attempt to minimize the discrepancy between the desired goal state and one’s current state of affairs. However, these models suggest that a ruminative response is not always maladaptive. Rather, repetitive attention to blocked goals can then either help or hinder an individual’s chances at minimizing goal state discrepancies, depending on how attention is focused during ruminative thought [10]. Specifically, studies using the Ruminative Responses Scale (RRS; [5,11]) have shown that higher scores on the Brooding subscale, which taps the tendency to passively “brood” over one’s problems and mood (e.g., “What am I doing to deserve this?”), relates to attentional biases toward [12], and difficulty disengaging from negative information [13]. In contrast, scoring higher on the Reflection subscale, which taps the tendency to deliberately “reflect” on concrete means for problem solving (e.g., “I write down what I am thinking and analyze it.”), is more often associated with less distraction and interference by self-relevant stimuli [14], indicating that the tendency to reflect seems to direct attention more adaptively.

Past research also suggests that the extent to which rumination produces adaptive or maladaptive effects may depend on factors such as gender and length of time elapsing from the initial negative event. Early investigations of this cognitive style revealed that dysphoric women generally tend to ruminate more than men [5,15], and experience more adverse consequences for their affective and cognitive states as a result, including the onset of clinically-significant depression [11,16,17]. In addition, the effects of rumination may compound over time, even in healthy undergraduates. For example, trait measures of rumination predicted healthy undergraduates’ levels of anxiety and feelings of hopelessness 4 to 8 h after they received the grade on their most difficult midterm exam, regardless of the actual score [18]. Taken together, these findings suggest the effects of Brooding and Reflection may become more apparent the longer that a goal is blocked, and that females might be particularly vulnerable to these cumulative effects. To our knowledge, past studies have not yet examined how gender, along with the length of time over which negative outcomes are experienced, impact the relationship between different subtypes of rumination and goal-directed behavior.

In the present study, we examined these relationships within a scenario that might signal a blocked goal and elicit rumination for the typical college student—the failure to meet an important academic goal. To create this context in a laboratory setting, we used an academically-framed general knowledge task developed in our lab [19,20]. In this computer-adaptive task, which is designed to elicit a pre-set rate of academic failure, students first answer a large set of questions and receive both performance feedback (colored asterisk) and learning feedback (correct answer) after each response. A surprise retest of all the items they initially answered incorrectly is then administered one to two days later. Because students are not informed that it is necessary to learn the correct answers during first-test, their rate of error correction on the retest provides a sense of how well they are able to incidentally learn from and use this feedback to rebound from their initial failures.

In addition to capturing behavioral measures of performance on the first-test and retest, we measured ERP responses to the performance and learning feedback to capture underlying mechanisms for how ruminative tendencies might influence rebound from failure. Our analysis of performance feedback focused on two waveforms: the medial frontal feedback-related negativity (FRN), which exhibits a relatively sharp deflection maximally at ~250 ms post-feedback [21,22], and the posteriorly maximal late positive potential (LPP), which typically begins about 400 ms post-stimulus [23,24]. The time course of these waveforms suggests that the FRN reflects an early, rapid, and relatively automatic detection of outcomes that are more negative (worse) than expected [22,25], whereas the LPP indexes later, more sustained attentional processes that are modulated by subjective levels of emotional arousal, regardless of emotional valence [26]. Furthermore, the LPP appears to persist even after the offset of the evoking stimulus [27], indicating that it can index not only externally-focused visual attention, but also motivated attention to an internal representation of an arousing stimulus.

Although the FRN has been studied extensively with regard to error feedback processing [21,22], the LPP has traditionally been studied in response to affective picture stimuli [27,28], rather than to feedback stimuli. However, in a recent study that used a math variant of the feedback-based learning task employed here [29], both the FRN and the LPP were observed following negative feedback and both influenced subsequent error correction, but in different ways. Specifically, Mangels and colleagues [29] found that for females who took the math test in a context of stereotype threat, a larger FRN was associated with poorer rebound from failure on the retest due to active avoidance of learning opportunities (*i.e.*, vigilance-avoidance pattern). In contrast, a larger LPP to negative feedback was associated not with avoidance, but with shallower processing of the learning opportunities, suggesting that working memory resources were not fully allocated toward task-relevant information and rather, might have been directed toward rumination on threat-related internal thoughts [30,31].

Taken together, these results would suggest that the LPP, even more so than the FRN, might be a prime candidate for modulation by trait rumination in the present study. Brooding should up-regulate arousal to negative feedback and, correspondingly, enhance LPP amplitudes, whereas Reflection should down-regulate this response, thereby attenuating the LPP. It is less clear, however, whether and how the FRN to negative feedback will be modulated by trait rumination. Even though recent evidence suggests that the FRN may partially index the affective response to negative outcomes (e.g., [32]), rumination is typically conceptualized as a *reactive* response occurring after negative outcomes. Thus, rumination may modulate processes occurring after the early error detection processes associated with the FRN. However, it is also possible that the FRN might be enhanced for subjects high in Brooding, who may have developed a habitual sensitivity to any context in which error signals might occur (e.g., [33]). This sensitivity might be especially evident in situations, such as the current task, where error events are repetitive, leading brooders to focus on passive and abstract construal of these events (*i.e.*, [34]). In contrast, a tendency toward more reflective rumination may produce an attenuation of the FRN (e.g., [35]). Finally, it is possible that habitual brooders and reflectors do not perceive repeated negative outcomes (e.g., failures) to be overly surprising, but rather have come to expect them. To the extent that the FRN is modulated by expectancy [22], this would mean that it may be attenuated for all ruminators—and given the prevalence of negative feedback in the task, this reduction in FRN amplitude may become even more evident as the task progresses.

Turning to the learning feedback processing, we focused this analysis on a group of sustained negative-going waveforms over inferior aspects of fronto-temporal and parietal-occipital regions. These electrode sites, which correspond to key regions of the semantic network (for review see [36]), have previously been shown to predict successful encoding of the correct answer in similar versions of this task [19,20]. Specifically, activity recorded at these sites during the initial presentation of the correct answer (*i.e.*, first-test) is more negative-going for answers that subjects later correctly recall on the subsequent retest, compared to those they later forget (*i.e.*, a difference due to memory; [37]). ERP studies have previously shown that activity at these sites is associated with activation of semantic representations (*i.e.*, a sustained negative-going waveform over fronto-temporal sites; [20,38,39,40]), and may be particularly important for deep conceptual and elaborative encoding of verbal information [20,41,42,43]. Thus, to the extent that Brooding or Reflection impacts the ability to correct errors, it may be through modulation of these encoding-related processes.

In sum, the aim of the present study was to examine whether trait tendencies toward Brooding and Reflection affected the feedback-based correction of errors in an academically-relevant general knowledge task. To understand the mechanisms associated with any effects on this goal-directed behavior, we investigated both overt measures of performance-related thoughts and feelings through subjective self-report, and covert measures of attention and arousal to errors using established ERP waveforms. We hypothesized that Brooding would be associated with poorer error correction on the retest, increased negative thoughts and feelings, and the up-regulation of arousal to these error signals (LPP). In contrast, Reflection would lead to preservation (or enhancement) of error correction, decreased negative thoughts and feelings about task performance, and correspondingly, an attenuated LPP to negative feedback. The predictions regarding the FRN were more complex and exploratory, as it is not clear whether and how this early component would be sensitive to conscious, reactive rumination processes. Finally, by dividing this lengthy task into four blocks and comparing across them, we examined how these behavioral and physiological responses changed as subjects repeatedly and relentlessly received high rates of negative feedback regardless of efforts to improve. We hypothesized that any differential effects of Brooding and Reflection should become enhanced as the task progressed, particularly for females, who also might simply be more sensitive to the effects of rumination overall. Thus, this investigation will add to a growing dialogue on how cognition and emotion interact with moderating variables, such as gender and time, to bias attention and influence goal-directed behavior in practical, real-world situations.

## 2. Method

### 2.1. Subjects

Fifty-four subjects (27 females) were recruited from a larger cohort of Baruch College undergraduate students who had completed a prescreening session that included administration of the Ruminative Responses Scale (RRS; [11]), as well as a series of questions assessing eligibility for EEG. Subjects enrolled in the 2-day EEG study were 18 to 30 years of age (*M* = 20.43, *SEM* = 0.38), right-handed, and native speakers of English or fluent by age six. All had normal or corrected-to-normal vision and hearing, and no self-reported history of neurological, psychological, or substance abuse disorders. Because the average time that elapsed between the prescreening session and the first day of the EEG study was 38.21 days (*SEM* = 2.04), all subjects completed the RRS again in a pre-test survey upon returning for EEG, and this latter score was used to predict electrophysiological and behavioral responses in the current study. All subjects scored less than 19 (*i.e.*, cutoff for moderate depression) on the Beck Depression Inventory II (BDI-II; [44]) in the pre-test survey on the day of EEG testing. Subjects received either monetary compensation at the rate of $10/h (65%), or a combination of course credit and monetary compensation (35%), for participation. A bonus incentive of $10 or 1 h of course credit was offered to all subjects who returned for the second day of testing.

Some subjects were excluded because critical aspects of their data did not conform to pre-determined standards. Three females had RRS scores that varied widely between the initial prescreen session and the latter pre-test survey (*i.e.*, greater than 2 SDs away from the sample mean difference), generating uncertainty that they completed one or both questionnaires of this trait measure appropriately. Five subjects (two females) completed only three of four total first-test blocks in the time they had available. One male performed well above (55%) the pre-determined first-test accuracy rate (35%) despite titration efforts (see Design and Procedure section), and at retest evidenced error correction rates that were at ceiling, suggesting that the task was not as challenging for this individual as it was for others. One female used a confidence rating of 1 (lowest confidence rating) on 93% of first-test question items, casting doubt not only on how she made use of this scale, but even on her engagement in the task. Two males had excessive EEG noise and too few usable trials in critical conditions (see EEG Recording). Finally, two subjects (one female) had unusable data due to computer malfunction.

The final sample included 40 subjects (20 females). As seen in Table 1, male and female groups were evenly matched for age, years of education, and depressive mood state. Overall scores on the 22-item RRS scale, as well as the 5-item Brooding and 5-item Reflection subscales, were highly similar to those originally reported by Treynor *et al.* [11], as well as other studies involving non-depressed undergraduate samples [45,46]. All rumination scores were equivalent between males and females (all *p* > 0.69). Brooding and Reflection subscores did not differ from one another, whether splitting by or collapsing across gender (all *p* > 0.38). Measures of internal consistency were good, (RRS: α = 0.91, Brooding: α = 0.79, Reflection: α = 0.81) and comparable to Treynor *et al.* [11].

### 2.2. Design and Procedure

#### 2.2.1. Overview

The study took place over two days. On the first day, subjects filled out a pre-test survey that contained both the RRS and BDI-II. After completing the survey packet, subjects were fitted with the EEG cap and answered 200 general knowledge questions divided into four blocks of 50 trials. A titration algorithm that used the normative difficulty of each question attempted to maintain a stable accuracy rate of 35% correct (*i.e.*, failure) for all subjects across all four blocks. This algorithm has been used previously in our research (for details about the titration algorithm, see [19]). Subjects were then asked to return 24–48 h later to complete a second set of general knowledge questions. Although subjects were given no further information about the type of questions they would receive on this second day, the questions were comprised solely of the items they had answered incorrectly on the first day. No EEG measures were captured on the second day of testing.

#### 2.2.2. First Day of Testing

**Figure 1:**
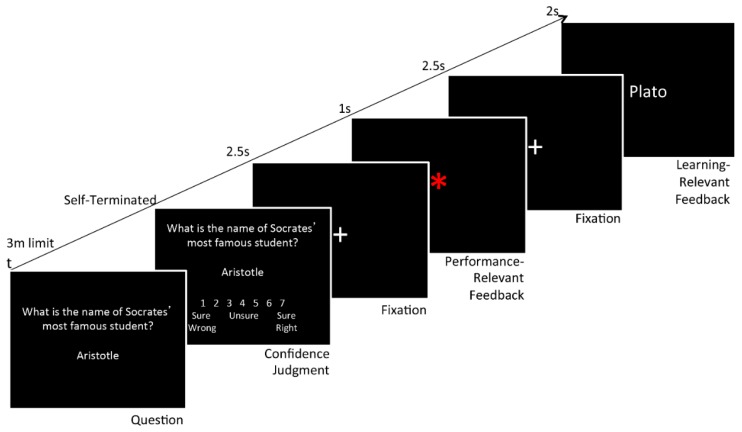
First-test trial structure. Example of a trial with an incorrect answer. If the answer had been correct, a green asterisk would have been shown.

The general knowledge trial sequence used on the first day is represented in Figure 1. Questions were presented one at a time and subjects were asked to provide their best response within a 3-min time limit. If they did not know an answer to a question they were prompted to type their best guess, or otherwise wait out the full duration of the time limit, after which they would be marked incorrect. Stimuli were taken from a pool of 434 general knowledge question-answer pairings tapping a wide variety of academic domains, including the natural and physical sciences, U.S. and world history, music and art history, literature, geography, and religion. All correct answers were one word, 3–12 letters in length. The mean difficulty of all questions in the pool was 35% correct, as specified from a norming study previously undertaken to determine the free response accuracy rate of each question within the Baruch College undergraduate population. In that study, each correct answer was rated as being a familiar word to at least 95% of the normative sample.

After submitting their response, subjects rated their confidence in their response on a 1–7 scale, where selecting 1 indicated “sure wrong”, 7 indicated “sure right”, and 4 indicated being “unsure” about the accuracy of their response. In the feedback sequence that followed, they were presented first with a blank screen for 500 ms, followed by a fixation crosshair for 2.5 s. Then, 1 s of performance-relevant feedback was given both visually and aurally in the pairing of a green-colored asterisk and a high tone for correct responses (*i.e.*, positive performance feedback), or a red-colored asterisk and a low tone for incorrect responses (*i.e.*, negative performance feedback). After another 2.5 s fixation point, learning-relevant feedback was presented for 2 s, consisting of the correct answer. We intentionally used relatively long pre-feedback wait periods (2.5 s) to allow time for subjects to turn attention inward toward any ruminative thoughts that might be generated both before and after the performance feedback (e.g., [47]). We expected that this would increase the likelihood of finding any effects of trait rumination on the processing of performance or learning feedback.

In order to gain insight into subjective experiences throughout the task, we asked subjects to answer four short post-block surveys about the thoughts (“How many recurring negative thoughts did you experience in the block of questions you just attempted?”) and feelings (“In this block of questions, whenever you made an error, how unpleasant or pleasant did you feel?”) they encountered in each block. All questions were rated on a 1-to-9 Likert scale, with 1 reflecting the negative or low end of the subjective experience, 9 reflecting the positive or high end, and 5 indicating an experiential midpoint (*i.e.*, neutrality). At the conclusion of the first day of testing, the EEG cap was removed and subjects were asked to return within 24–48 h to complete a second set of general knowledge questions.

#### 2.2.3. Second Day of Testing

The retest trial sequence differed from that of first-test in that the feedback was presented all at once in order to minimize the amount of time subjects had to spend in the lab on the second day of testing. Correct answers appeared in green text and were paired with a high tone, and incorrect answers appeared in red text and were paired with a low tone. This combined feedback was presented immediately following subjects’ confidence ratings and lasted for 1 s. All re-queried questions were presented in one large retest block. Question order was randomized with the exception that, to decrease variability in study-test delay and preserve some aspects of test context, all questions from first-test blocks 1 and 2 were binned and re-queried before the questions from first-test blocks 3 and 4. Only at the outset of this second day of testing were subjects made aware that they were being retested on a subset of questions they had answered on the first day. Upon completion of these questions, all subjects reported being surprised by the retest.

### 2.3. EEG Recording

EEG was recorded continuously using a sintered Ag/AgCl 64-electrode Quick-Cap. The analog signal was amplified using Neuroscan Synamps 2 and converted to digital at a rate of 500 Hz with a bandpass of DC-100 Hz. Impedance was kept below 11 kΩ. EEG was initially referenced to Cz during recording, and afterwards converted to an average reference off-line. We used 4–6 PCA-derived ocular components to compensate for blinks and other eye movement artifacts. The continuous EEG data was cut into epochs separately for performance- and learning-relevant feedback and then time-locked to the onset for each type (−100 ms to 1000 ms). After conducting baseline correction to the 100 ms window preceding the feedback stimulus, we rejected any epochs containing excessive noise (±100 µV) and averaged all remaining epochs for event-related potential (ERP) analysis. Single-subject averages were generated at the overall and block levels. Prior to averaging, both low-pass (35 Hz) and high-pass (0.12 Hz) filters were applied to the EEG data.

### 2.4. Data Analysis

#### 2.4.1. Overview

Our study focuses on the relationship between Brooding and Reflection RRS subscores (hereafter referred to as Brooding and Reflection) and measures of memory, subjective experience, and ERP waveforms. To this aim, we used a customized general linear model to test these relationships by entering Brooding and Reflection RRS subscores as simultaneous covariates (*i.e.*, predictor variables) in a 2 (gender) by 4 (block) mixed-measures Analysis of Covariance (ANCOVA) and rendering parameter estimates (converted to standardized beta coefficients) for individual difference measures. We also included all 2- and 3-way interaction terms (including those with Brooding or Reflection) in the customized model for the purpose of testing how the two subtypes of rumination may have interacted with gender and/or block to influence the measures of interest.

As in previous studies of rumination in non-depressed samples, our customized ANCOVAs included not only Brooding and Reflection, but also BDI-II scores, entered as another simultaneous covariate (see [48,49]). Indeed, although all students had BDI-II scores under the threshold for moderate depression, BDI-II was correlated with both Brooding (*r* = 0.38, *p* < 0.05) and Reflection (*r* = 0.47, *p* < 0.005). Including BDI-II as a simultaneous predictor variable helped to ensure that we were evaluating the effects of trait rumination after contributions from depression had been removed [45,46,48,49,50]. The effect of BDI-II was also evaluated as a main effect, as well as across all 2- and 3-way interactions involving gender and/or block factors. Because depression is not of central interest to this study, however, we will report relationships involving BDI-II in Supplemental Section S1.

All dependent measures were adjusted to control for the potential confounding influence of first-test accuracy prior to their inclusion in the customized ANCOVAs, as we typically do for this paradigm [19,29]. We adjust for first-test performance because, even after titration, individuals who perform better at first-test typically also perform better at retest, perhaps because they have slightly fewer items to correct, or because they have a stronger knowledge base. To control for this variable, we regressed first-test accuracy from our dependent variables prior to inserting those measures into our analyses. This approach permitted use of the properly adjusted block scores in our ANCOVAs and maximized degrees of freedom when conducting our customized general linear models.

Across all analyses, we used the conventional alpha level of *p* ≤ 0.05 as the criterion for significance, but also report marginally significant findings (0.05 < *p* ≤ 0.10), specifying effect sizes in all cases. Where necessary, Greenhouse-Geisser corrections were used for violations of sphericity. In addition, where appropriate, linear trend analyses were conducted across the within-subjects factor of block to determine how any particular dependent measure may have changed over time. Any *post hoc* explorations of significant main effects or interactions were carried out using the Holm-Bonferroni procedure for corrections for multiple comparisons [51].

#### 2.4.2. ERP Responses to Performance-Relevant Feedback

ERPs for performance-relevant feedback were averaged according to first-test accuracy (average number of correct trials: 63.78, *SEM* = 1.19; average number of incorrect trials: 116.38, *SEM* = 1.52). All subjects had at least 10 trials in each of the four blocks for both correct and incorrect responses, thus permitting ample signal-to-noise ratios for analysis of the FRN and LPP at the block level [52]. There were no gender differences in the amount of trials available for the analysis of the ERP response to negative performance, whether overall or by block (all *F* < 1.70, all *p* > 0.17).

##### The Feedback-Related Negativity (FRN)

Visual inspection of grand average waveforms corroborated the existence of an FRN that was visibly larger for errors, most prominently over midline anterior scalp regions (Fz; [20,53]). To measure this waveform, we first identified the largest negativity from 200–350 ms post negative feedback at Fz (*i.e.*, the peak), and then measured the average amplitude within a 30 ms time window centered over that peak (e.g., [54,55]). We refer to this ERP response to negative feedback as the FRN_neg_. Although peak-picking the FRN_neg_ was relatively straightforward, most subjects lacked a definitive FRN to positive feedback, and thus, in an attempt to reduce sampling error, we opted to measure this ERP response (FRN_pos_) at the same peak latency that was used for the FRN_neg_.

Consistent with past research [56], the FRN_neg_ was more negative-going than the FRN_pos_ regardless of gender or block, although both the FRN_neg_ and FRN_pos_ became more negative-going as the task progressed (see Supplemental Section S2.1). Because our study focuses on the neural and behavioral response to negative feedback, our primary RRS analyses focused on the FRN_neg_ and, in order to address the downward trend of both FRN waveforms, the FRN_diff_ (*i.e.*, FRN_neg_−FRN_pos_).

##### The Late Positive Potential (LPP)

Visual inspection of grand average waveforms and topographies corroborated the existence of the LPP over multiple posterior-superior sites (see also [57]). To simplify our investigation of RRS effects on the LPP, we focused on the LPP to negative feedback (LPP_neg_), and analyzed the mean amplitude averaged over central-parietal and parietal scalp regions (CP1/CPz/CP2, P3/Pz/P4; see Supplemental Section S2.2 for analyses relating to use of this averaged electrode cluster, as well as comparison of the LPP_neg_ and LPP_pos_). We analyzed this averaged amplitude within two separate time windows: 400–600 ms and 600–1000 ms (see also [58]). We hereafter refer to these two windows as the early and late LPP, respectively.

#### 2.4.3. ERP Response to Learning-Relevant Feedback

Evaluation of the ERPs to learning-relevant feedback (*i.e.*, the correct answer) focused on a posterior-inferior cluster (TP9/TP10, CB1/CB2, and O1/O2) and a left-inferior cluster (F7, FT9, T7, TP9, CB1, O1) from 500 to 1000 ms, post-stimulus onset, reflecting the spatiotemporal distribution of “difference due to memory (Dm)” effects in the present study (see Supplemental Section S2.3.), which were consistent with those typically found for this paradigm [19,20]. Because we did not have sufficient trials to conduct traditional Dm analyses for each block, however, our main analyses involving block and RRS subscores used the overall learning-feedback ERP after errors (*i.e.*, collapsing across items subsequently recalled and not recalled), which we refer to as the Learning Event-Related Negativity (LERN; [20]).

We created both the posterior-inferior and left-inferior LERN ERPs by averaging first-test errors, regardless of whether those errors were later corrected or not during the retest (average number of trials: 112.33, *SEM* = 1.73). All subjects had at least 18 trials in each block, and no gender differences emerged in the amount of trials used for ERP analysis, whether overall or by block (all *F* < 0.45, all *p* > 0.51) (The proportion of later corrected and not corrected LERN trials that survived artifact rejection was not statistically different from the proportion of corrected and not corrected trials measured behaviorally at retest within any block, whether overall, or as a function of gender (all *t* < 1.30, all *p* > 0.20), lending confidence to the view that the LERN should accurately scale with actual memory behavior.). The LERN from 500 to 1000 ms at these locations was strongly correlated with remedial behavior for females (Posterior cluster: *r* = −0.57, *p* < 0.01; Left cluster: *r* = −0.65, *p* < 0.005), although surprisingly for males, this effect was only marginal in the left-inferior cluster: *r* = −0.40, *p* = 0.08, while in the posterior-inferior cluster it was not significant: *r* = −0.32, *p* = 0.17 (though the relationship was in the expected direction).

## 3. Results

### 3.1. Memory Performance

Table 2 shows both the proportions of items initially correct at first-test (where the target accuracy for the titration algorithm was 0.35) and the proportions of items initially incorrect at first-test that were later corrected on the subsequent surprise retest. Both overall performance (task-wide) and performance during each 50-item block are shown as a function of gender.

#### 3.1.1. First-Test Accuracy

As shown in Table 2, males outperformed females at first-test despite titration efforts (main effect of gender, *F*(1, 32) = 5.31, *p* < 0.05, η*_p_*^2^ = 0.14). Indeed, males’ overall performance significantly exceeded the target titration value, as verified by a one-group t-test against 0.35, *t*(19) = 2.20, *p* < 0.05, whereas females’ performance was within range of titration, *t*(19) = 1.36, *p* = 0.19. Nonetheless, the lack of a block effect, or a gender by block interaction, indicated that within each gender, the titration was effective in keeping first-test performance stable (all *F* < 0.87, all *p* > 0.46).

**Figure 2:**
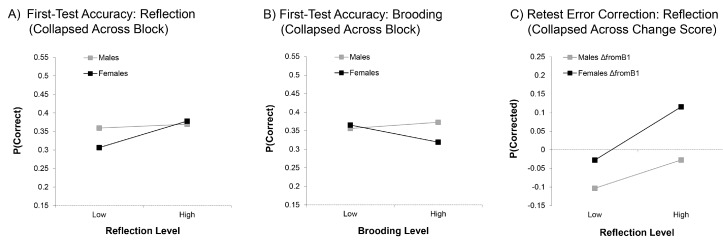
Memory performance as a function of RRS and gender. Effects of Reflection (**A**) and Brooding (**B**) on the proportion of first-test questions answered correctly across all blocks; (**C**) Effects of Reflection on change in error correction between Block 1 and subsequent blocks.

Males’ superior performance at first-test was related to neither Brooding nor Reflection, whereas both RRS subscores predicted females’ performance in some way. Specifically, we found an overall effect of Reflection, *F*(1, 32) = 7.93, *p* < 0.01, η*_p_*^2^ = 0.20, that was qualified by both a gender by Reflection interaction, *F*(1, 32) = 4.49, *p* < 0.05, η*_p_*^2^ = 0.12, and a marginal 3-way interaction additionally involving block, *F*(2.11, 67.45) = 2.65, ε = 0.70, *p* = 0.08, η*_p_*^2^ = 0.08. Focusing on the significant 2-way interaction, we found that Reflection predicted significantly better overall performance at first-test in females, β = 0.80, *t* = 3.02, *p* < 0.01, η*_p_*^2^ = 0.22 (see Figure 2A), but not males, β = 0.21, *t* = 0.61, *p* = 0.55, η*_p_*^2^ = 0.01.

The influence of Brooding on first-test performance also differed across gender, *F*(1, 32) = 4.91, *p* < 0.05, η*_p_*^2^ = 0.13, but not block. Females with higher Brooding scores had marginally poorer performance throughout the task, β = − 0.51, *t* = 1.94, *p* = 0.06, η*_p_*^2^ = 0.11. There was no significant relationship between Brooding and first-test performance in males, β = 0.36, *t* = 1.09, *p* = 0.28, η*_p_*^2^ = 0.04, although the finding that it was in the opposing direction to females likely contributed to the strength of the 2-way interaction (see Figure 2B).

As can be seen in the Figure 2A,B, females who were lower on trait Reflection and higher on trait Brooding exhibited lower retrieval success at first-test compared to males. For males, in contrast, RRS subscores had little bearing on performance.

#### 3.1.2. Error Correction

Despite females’ poorer first-test performance overall, they outperformed males on the retest, an effect that was right at the conventional level of significance, *F*(1, 32) = 4.11, *p* = 0.05, η*_p_*^2^ = 0.11 (see Table 2). Time in the task (*i.e.*, block) also had an overall effect on error correction, *F*(3, 96) = 2.90, *p* < 0.05, η*_p_*^2^ = 0.08, such that correction rates decreased over the course of the task. However, both of these main effects were qualified by a significant gender by block interaction, *F*(3, 96) = 3.39, *p* < 0.05, η*_p_*^2^ = 0.10. Exploration of this interaction indicated that while males corrected fewer errors as the task progressed, as evidenced by a significant downward linear trend across block, *F*(1, 16) = 6.24, *p* < 0.05, η*_p_*^2^ = 0.28 (see Table 2), females exhibited fairly stable error correction across the task. As a result, the females’ relative advantage over males on error correction appeared to increase as the task progressed (*i.e.*, in Blocks 3 and 4), although none of the *post hoc* comparisons of gender differences at each block survived Holm-Bonferroni corrections.

When then considering the effects of RRS on error correction, we found no effects of Brooding, either overall or as a function of gender or block, in contrast to our predictions. More in support of our hypotheses, however, significant effects of Reflection emerged that appeared to differ across blocks, *F*(3, 96) = 2.75, *p* < 0.05, η*_p_*^2^ = 0.08, though not between gender. When unpacking the interaction as a function of block, however, none of the parameter estimates survived Holm-Bonferroni corrections. Nonetheless, it is worth noting that numerically, the parameter estimates in Block 1 demonstrated a slightly negative relationship between Reflection and error correction (Block 1: β = −0.13), whereas the parameter estimates in the three subsequent blocks were all in the *positive* direction (Block 2: β = 0.45; Block 3: β = 0.10; Block 4: β = 0.13). Thus, it is possible that these opposing, though non-significant effects drove the significant interaction of Reflection with block.

To explore this possibility further, we calculated the change in error correction between the initial block (Block 1) and each of the subsequent blocks. In this way, Block 1 was treated as a baseline to produce the following change scores: the change from Block 1 to Block 2 (*i.e.*, ΔB1-to-B2), Block 1 to Block 3 (*i.e.*, ΔB1-to-B3), and Block 1 to Block 4 (*i.e.*, ΔB1-to-B4). We then used these change scores as a 3-level “change” factor (to replace the 4-level “block” factor) in a 2 (gender) by 3 (change) mixed-measures ANCOVA that included the customized 2- and 3-way interaction terms. Using these change scores, we found an overall gender effect, *F*(1, 32) = 9.30, *p* < 0.01, η*_p_*^2^ = 0.23, where males showed a change for the worse in performance from Block 1 relative to females, similar to what was described by the block analysis above.

In addition, we found a main effect of change, *F*(2, 64) = 4.23, *p* < 0.05, η*_p_*^2^ = 0.12, but no interaction between gender and change. *Post hoc* testing of the change effect indicated that subjects overall demonstrated a greater change for the worse (*i.e.*, decrease) in error correction rates during the later two blocks relative to the ΔB1-to-B2 period (*vs*. ΔB1-to-B3: *p* < 0.05; *vs.* ΔB1-to-B4: *p* < 0.05). The change in error correction in these latter two periods did not differ from each another (*p* = 0.89). More importantly, however, a main effect of Reflection emerged, *F*(1, 32) = 4.10, *p* = 0.05, η*_p_*^2^ = 0.11, with a positive parameter estimate (β = 0.37).

Figure 2C illustrates the simple effects for this relationship, showing that change in error correction across the task benefitted from a greater tendency toward Reflection in both genders. For females, higher Reflection scores were associated with relative improvement in error correction in later blocks relative to Block 1, whereas lower Reflection scores were associated with little change in either direction. For males, however, lower Reflection scores were associated with decline across the task, whereas for those with higher Reflection scores, little change was evident.

### 3.2. Subjective Experiences

The extent to which male and female subjects reported experiencing recurring negative thoughts (RNTs) and negative feelings after errors (FAEs) is reported in Table 3, both overall and over the course of the four task blocks.

#### 3.2.1. Recurring Negative Thoughts (RNTs)

As shown in Table 3, RNTs lessened somewhat over the course of the task for both females and males, as supported by a marginal effect of block, *F*(3, 96) = 2.28, *p* = 0.09, η*_p_*^2^ = 0.07, and a significant linear trend analysis, *F*(1, 32) = 4.95, *p* < 0.05, η*_p_*^2^ = 0.13. Although this downward direction was unexpected, analysis of the effect of RRS on RNTs indicated that this effect had to be considered in light of moderation by Brooding, Reflection, and gender.

In line with our predictions that the effects of Brooding might differ as a function of gender or time in the task, we found a significant 3-way interaction amongst Brooding, gender, and block, *F*(3, 96) = 5.32, *p* < 0.005, η*_p_*^2^ = 0.14. Although *post hoc* analysis of the source of this interaction did not find any parameter estimates that survived Holm-Bonferroni corrections, as with the analysis of error correction, further inspection of these estimates demonstrated that for females, the relationship between Brooding and RNTs became increasingly positive across the four blocks (Block 1: β = 0.04, Block 2: β = 0.21, Block 3: β = 0.56, Block 4: β = 0.59), whereas for males it started out as positive-going in Blocks 1 (β = 0.11) and 2 (β = 0.12), but became negative-going in Blocks 3 (β = −0.29) and 4 (β = −0.29). These apparent gender differences in the direction of Brooding effects on RNTs was further substantiated by a significant interaction in the linear trend across blocks as a function of gender, *F*(1, 32) = 10.74, *p* < 0.005, η*_p_*^2^ = 0.25.

**Figure 3:**
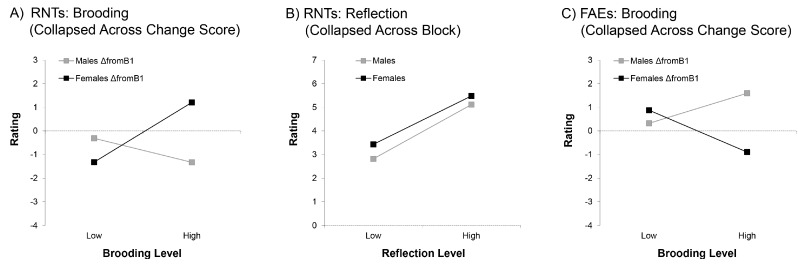
First-test subjective experiences as a function of RRS and gender. (**A**) Effects of Brooding on change in RNTs between Block 1 and subsequent blocks; (**B**) Effects of Reflection on RNTs collapsed over four blocks; (**C**) Effects of Brooding on change in FAEs between Block 1 and subsequent blocks.

Given these apparent differences in Brooding effects in the early compared to later blocks, we again applied our change score approach to this analysis. We found both a 2-way interaction between gender and Brooding, *F*(1, 32) = 5.45, *p* < 0.05, η*_p_*^2^ = 0.17, and a 3-way interaction of gender, change score and Brooding, *F*(2, 64) = 5.21, *p* < 0.01, η*_p_*^2^ = 0.14. When *post hoc* investigation of the 3-way interaction again did not reveal any parameter estimates that survived Holm-Bonferroni corrections, we turned our attention to closer examination of the 2-way interaction. Here, as illustrated in Figure 3A, we found evidence that Brooding marginally increased females’ RNTs throughout the task compared to Block 1, β = 0.61, *p* = 0.06, *t* = 1.98, η*_p_*^2^ = 0.11. In contrast, it had no significant effect on males’ RNT scores, which numerically demonstrated a negative relationship, β = −0.38, *t* = 1.23, *p* = 0.23, η*_p_*^2^ = 0.05.

Interestingly, in the block analysis, Reflection was also a significant predictor of increased RNTs (β = 0.53; see Figure 3B), indicating that both subtypes of rumination resulted in the generation of negative thoughts about the task, *F*(1, 32) = 6.95, *p* < 0.05, η*_p_*^2^ = 0.18. This effect did not interact with gender and/or block (all *F* < 0.50, all *p* > 0.67). The fact that this effect was consistently found across all four blocks was substantiated by the lack of any effect of Reflection when analyzing change scores relative to Block 1 (all *F* < 0.52, all *p* > 0.48).

#### 3.2.2. Feelings After Errors (FAEs)

Males’ FAEs started off as somewhat unpleasant in Block 1, but became more neutral as the task progressed (see Table 3). Females on the other hand, while reporting roughly similar FAEs to males in Block 1, maintained these somewhat unpleasant FAEs throughout the task. These gender differences were supported by a significant gender by block interaction, *F*(3, 96) = 3.57, *p* < 0.05, η*_p_*^2^ = 0.10, that superseded an overall block effect, *F*(3, 96) = 3.89, *p* < 0.05, η*_p_*^2^ = 0.11. This interaction appeared to be related to the fact that for males, there was a significant upward linear trend for FAEs, *F*(1, 16) = 19.04, *p* < 0.001, η*_p_*^2^ = 0.54, whereas for females, there appeared to be no such change, *F*(1, 16) = 0.04, *p* = 0.84, η*_p_*^2^ = 0.003.

Reflection did not appear to have any influence on subjects’ feeling about their errors, either overall or as a function of gender or block, all *F* < 0.52, all *p* > 0.62. In contrast, Brooding did influence FAEs, although this effect differed as a function of block and gender, as indicated by a significant 3-way interaction, *F*(3, 96) = 2.93, *p* < 0.05, η*_p_*^2^ = 0.08. Although none of the *post hoc* evaluations of parameter estimates revealed significant effects following Holm-Bonferroni corrections, further inspection suggested males and females began to diverge in how Brooding affected their feelings about errors after Block 1. Whereas Males exhibited a negative-going coefficient in Block 1 (β = −0.50), in the subsequent three blocks, this relationship reversed to be positive-going (Block 2: β = 0.20, Block 3: β = 0.17, Block 4: β = 0.30). Females started Block 1 with a near-zero coefficient (β = −0.07), which then became more negative-going during the remainder of the task (Block 2: β = −0.56, Block 3: β = −0.48, Block 4: β = −0.50).

Once again, these apparent contrasts in the direction of change in FAEs for males and females following Block 1 motivated us to shift our analysis to change scores. We found an overall effect of gender on change scores, *F*(1, 32) = 6.46, *p* < 0.05, η*_p_*^2^ = 0.17, consistent with what was found above when analyzing all four blocks. Females felt more negatively about their errors in later blocks compared to Block 1, whereas males felt more neutral. However, these opposing effects for males and females were exacerbated by Brooding, as revealed in a significant gender by Brooding interaction, *F*(1, 32) = 7.30, *p* < 0.05, η*_p_*^2^ = 0.19. As shown in Figure 3C, males and females who were lower in Brooding showed relatively similar levels of change across the task; but for males higher in Brooding, FAEs became more positive over the task relative to Block 1, β = 0.59, *t* = 2.07, *p* < 0.05, η*_p_*^2^ = 0.12, while for females higher in Brooding, FAEs became marginally more negative, β = −0.59, *t* = 1.87, *p* = 0.07, η*_p_*^2^ = 0.10.

### 3.3. Event-Related Potentials (ERPs)

Amplitudes of the waveforms of interest for the performance feedback (FRN, early and late LPP) and learning feedback (posterior and left hemisphere LERN) are shown in Table 4 for each gender, both over the entire task and as a function of block.

#### 3.3.1. Feedback Related Negativity (FRN)

**Figure 4:**
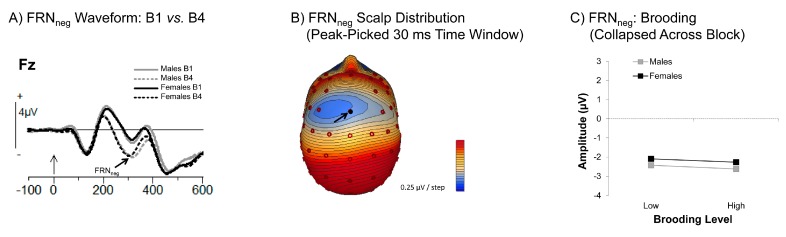
Feedback-Related Negativity in response to errors (*i.e.*, FRN_neg_). (**A**) FRN_neg_ grand mean waveforms plotted at Fz for the first (B1) and last (B4) blocks, as a function of gender; (**B**) Scalp distribution of the FRN_neg_ for all subjects, collapsed over all four blocks. The arrow points to Fz, which is highlighted in black; (**C**) Null effects of Brooding on FRN_neg_ amplitude.

Figure 4A shows the FRN_neg_ in the first and last block, providing an illustration of the negative trend for this waveform, in both genders, as the task progressed (see also Supplemental Section S2.1). The scalp topography of this waveform, collapsed across the task, is shown in Figure 4B.

Neither Brooding nor Reflection RRS subscores were predictive of the FRN_neg_, either overall, or as a function of gender and/or block (all *F* < 1.36, all *p* > 0.26, see Figure 4C illustrating the null findings for Brooding only). Similarly, Brooding and Reflection also did not predict amplitude of the FRN difference wave (FRN_diff_; all *F* < 1.21, all *p* > 0.28; see Supplemental Section S2.1). An exploration of the effects of Brooding and Reflection on FRN_neg_ and FRN_diff_ change scores also yielded null effects (all *F* < 2.40, all *p* > 0.13).

#### 3.3.2. Late Positive Potential (LPP)

**Figure 5:**
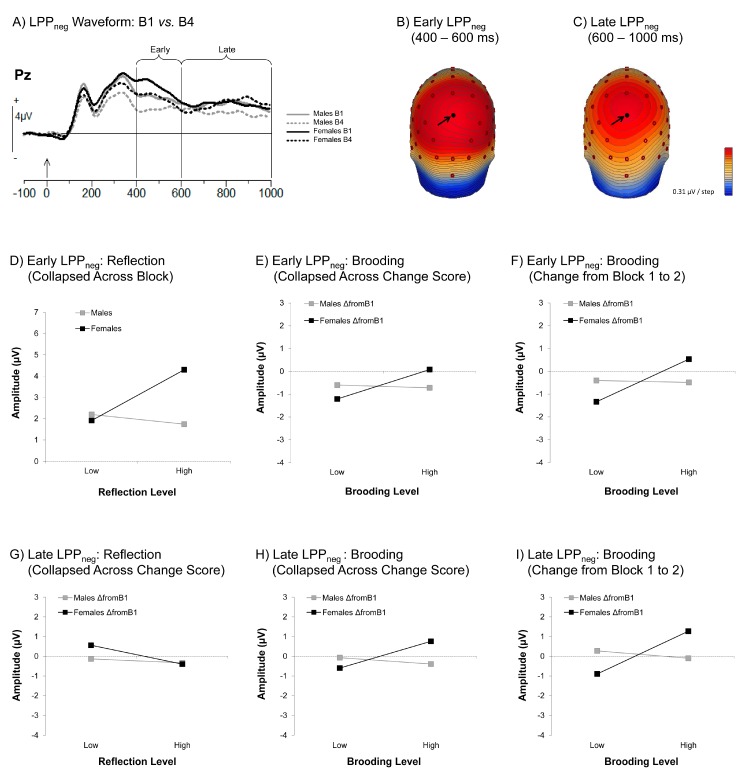
Late Positive Potential in response to errors (*i.e.*, LPP_neg_) as a function of RRS and gender. (**A**) Early (400–600 ms) and Late (600–1000 ms) LPP_neg_ grand mean waveforms plotted at Pz for the first (B1) and last (B4) blocks. (**B**,**C**) Scalp distribution of the LPP_neg_ in the early and late periods, respectively, for all subjects, collapsed across all four blocks. The arrows point to Pz, which is highlighted in black. (**D**) Effects of Reflection on early LPP_neg_ amplitude. Effects of Brooding on change in early LPP_neg_ amplitudes between Block 1 and subsequent blocks (**E**), and during the change from Blocks 1 to 2 only (**F**). (**G**) Main effect of Reflection on late LPP_neg_ amplitude. Effects of Brooding on change in late LPP_neg_ amplitudes between Block 1 and subsequent blocks (**H**), and during the change from Blocks 1 to 2 only (**I**).

Figure 5A shows the LPP to negative feedback (LPP_neg_) as a function of gender and block (Block 1 *vs.* 4) at the midline parietal electrode (Pz), a representative site that illustrates effects on both the early (400–600 ms) and later (600–1000 ms) aspects of this waveform (Figure 5B,C; see also Supplemental Section S2.2).

##### The Early LPP_neg_

The amplitude of the early LPP_neg_ was larger overall for females than males, *F*(1, 32) = 8.13, *p* < 0.01, η*_p_*^2^ = 0.20. However, this gender difference was moderated by Reflection, *F*(1, 32) = 5.54, *p* < 0.05, η*_p_*^2^ = 0.15, in that the females with a greater tendency to Reflect demonstrated an enhanced early LPP_neg_, β = 0.63, *t* = 2.41, *p* < 0.05, η*_p_*^2^ = 0.15 (see Figure 5D). Assessment of this effect also indicated that females with lower Reflection scores exhibited an early LPP_neg_ similar to that of males, whose early LPP_neg_ did not differ as a function of Reflection.

Although this finding is consistent with the general prediction that the LPP_neg_ would be the ERP response particularly sensitive to rumination, particularly in females, it contrasts with our prediction that Reflection would down-regulate (reduce) this response. In addition, our prediction that this effect would interact with time in the task was not upheld, as there were no interactions involving both Reflection and block (all *F* < 1.86, all *p* > 0.14.). Indeed, no significant effects of Reflection were found when we conducted a change score analysis, further supporting the view that this gender-specific Reflection effect occurred consistently across the task, including as early as Block 1.

The relationship between Reflection and early LPP_neg_ amplitude in females did not appear to change across the task; however, we did find a significant overall effect of block, *F*(3, 96) = 12.33, *p* < 0.001, η*_p_*^2^ = 0.28, coupled with a significant downward linear trend, *F*(1, 32) = 25.92, *p* < 0.001, η*_p_*^2^ = 0.45 (see also Supplemental Section S2.2.1), supporting the impression that the early LPP_neg_ became attenuated as the task progressed (see also Table 4). In contrast to Reflection, however, Brooding interacted with this block effect, as well as gender, as indicated by a significant 3-way interaction, *F*(3, 96) = 3.98, *p* < 0.05, η*_p_*^2^ = 0.11. Specifically, exploration of parameter estimates uncovered a *negative* relationship between Brooding and early LPP_neg_ for females that was strongest in Block 1 (β = −0.52) and weakened across all remaining blocks (Block 2: β = −0.07, Block 3: β = −0.45, Block 4: β = −0.17), although no parameter estimates for individual blocks survived Holm-Bonferroni correction.

When we used change scores to explore this apparent weakening further, however, we found a marginal 2-way interaction of Brooding by gender, *F*(2, 64) = 3.95, *p* = 0.06, η*_p_*^2^ = 0.11, and a significant 3-way interaction of Brooding, gender, and change, *F*(2, 64) = 3.99, *p* < 0.05, η*_p_*^2^ = 0.11. *Post hoc* exploration of the 2-way interaction indicated that a greater tendency toward Brooding in females predicted less attenuation of the early LPP to error signals across all change periods compared to the Block 1 baseline period, β = 0.70, *t* = 2.17, *p* < 0.05, η*_p_*^2^ = 0.13 (Figure 5E). When pursuing the source of the 3-way interaction, we found that this effect was driven primarily by the ΔB1-to-B2 period, β = 0.83, *t* = 3.09, *p* < 0.05, η*_p_*^2^ = 0.23 (Figure 5F).

##### The Late LPP_neg_

When considering how RRS subscores related to the late LPP_neg_, we found evidence that both trait Reflection and Brooding influenced the amplitude of this waveform. However, whereas the effects of Brooding were largely similar across the early and late LPP_neg_, there were some important differences with regard to Reflection.

Effects involving both RRS subscales were found to differ as a function of both gender and block (Reflection, *F*(3, 96) = 2.69, *p* = 0.05, η*_p_*^2^ = 0.08; Brooding: *F*(3, 96) = 4.76, *p* < 0.005, η*_p_*^2^ = 0.13). These three-way interactions subsumed marginally significant 2-way interactions with both block (Brooding: *F*(3, 96) = 2.43, *p* = 0.07, η*_p_*^2^ = 0.07), and gender (Reflection: *F*(1, 32) = 3.84, *p* = 0.06, η*_p_*^2^ = 0.11; Brooding: *F*(1, 32) = 3.67, *p* = 0.06, η*_p_*^2^ = 0.10). To put these 3-way interactions with gender and block in perspective, we note that they occurred despite no overall effect of gender, or a 2-way interaction between gender and block (all *F* < 2.32, *p* > 0.14). As with the early LPP_neg_, however, there was still a significant main effect of block, *F*(3, 96) = 2.72, *p* < 0.05, η*_p_*^2^ = 0.08, corresponding to a significant downward linear trend in the amplitude of this waveform as the task progressed, *F*(1, 32) = 4.29, *p* < 0.05, η*_p_*^2^ = 0.12.

In prior analyses involving key variables, we have repeatedly found that the analysis of change scores produced a clearer understanding of the interactions involving time on task than those involving block; thus, with the goal of simplifying our analyses, we chose to move directly to change scores for analysis of the late LPP_neg_. In doing so, we found that both RRS subscales exhibited 3-way interactions with gender and change (Reflection: *F*(2, 64) = 3.18, *p* < 0.05, η*_p_*^2^ = 0.09; Brooding: *F*(2, 64) = 3.74, *p* < 0.05, η*_p_*^2^ = 0.11). In addition, we found a significant 2-way gender by Brooding interaction, *F*(1, 32) = 7.33, *p* < 0.05, η*_p_*^2^ = 0.19.

For Reflection, none of the *post hoc* comparisons examined in the process of unpacking the 3-way interaction reached significance after Holm-Bonferroni correction, with only the ΔB1-to-B2 parameter estimate for females approaching even marginal significance, β = −0.64, *t* = 2.45, *p* = 0.12, η*_p_*^2^ = 0.16. When coupled with a marginal main effect of Reflection, *F*(1, 32) = 3.30, *p* = 0.08, η*_p_*^2^ = 0.09, also showing a negative parameter estimate (β = −0.26; see Figure 5G), we can infer that the early and late LPP_neg_ were influenced by Reflection in different ways. In this later portion of the LPP_neg_, a tendency toward Reflection did not heighten the LPP response, and rather may have somewhat attenuated it.

The effects of Brooding on the late LPP_neg_ change scores, on the other hand, were more similar to the pattern found for the early LPP_neg_. Consistent with findings for the early LPP_neg_, exploration of the significant 2-way interaction indicated that females’ Brooding scores predicted less overall attenuation in the late LPP_neg_ compared to baseline, β = 0.72, *t* = 2.61, *p* < 0.05, η*_p_*^2^ = 0.18 (Figure 5H). When we additionally addressed the 3-way interaction indicating that this gender by Brooding interaction varied across change score, we found these effects were once again driven primarily by reduced attenuation in sustained attention to error signals during Block 2 relative to Block 1, β = 0.96, *t* = 3.67, *p* < 0.01, η*_p_*^2^ = 0.30 (Figure 5I).

#### 3.3.3. Learning Error Related Negativity (LERN)

**Figure 6:**
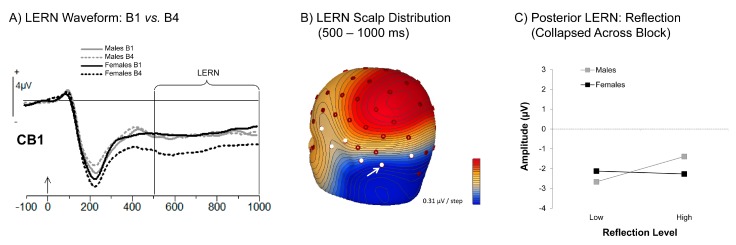
Learning Error-Related Negativity (LERN). (**A**) LERN grand mean waveforms plotted at CB1 for the first (B1) and last (B4) blocks, as a function of gender; (**B**) Scalp distribution of the LERN plotted for all subjects, collapsed across all four blocks. Left hemisphere electrodes included in the analysis are highlighted in white, with an arrow pointing to CB1; (**C**) Null effects of Reflection on Posterior LERN amplitude.

Figure 6A illustrates effects of gender and block (Block 1 *vs.* 4) on the LERN at CB1, a site at the convergence of both posterior and left hemisphere regions (see also Figure 6B). These regions were the most sensitive to overall subsequent memory effects (see Supplemental Section S2.3).

As illustrated by Figure 6A, females’ LERN became more negative-going subsequent to Block 1, whereas males evidenced no such change. This observation was corroborated by *post hoc* comparisons following significant gender by block interactions found at both the posterior-inferior electrode cluster, *F*(3, 96) = 5.32, *p* < 0.005, η*_p_*^2^ = 0.14, and left hemisphere electrode cluster, *F*(3, 96) = 3.64, *p* < 0.05, η*_p_*^2^ = 0.10. The finding that females’ Block 1 was different from all subsequent blocks was further substantiated by a change score analysis, which found only a main effect of gender, *F*(1, 32) = 10.43, *p* < 0.005, η*_p_*^2^ = 0.25.

In contrast to predictions that RRS might directly influence encoding of the correct answer, neither Brooding nor Reflection demonstrated any relationship to either the posterior-inferior or left hemisphere LERN waveforms (Brooding: all *F* < 0.09, all *p* > 0.76; Reflection: all *F* < 1.42, all *p* > 0.24; see Figure 6C illustrating the null findings with the left hemisphere LERN for Reflection only). Exploratory analyses using change scores also rendered no significant effects involving either RRS subscore in either region (all *F* < 1.45, all *p* > 0.24).

## 4. Discussion

The present study sought to investigate how cognition and emotion interact to influence the ability to learn and rebound from failure in the context of a challenging general knowledge task. Toward this end, undergraduates’ trait levels of Brooding and Reflection rumination were used to predict both subjective and event-related potential (ERP) responses to negative feedback, as well as the ability to use this feedback to correct errors on a subsequent surprise retest. We further explored whether any effects of rumination might be moderated by the gender of the student and the length of time the student experienced persistent failure in the task. These findings could serve to clarify the role that different subtypes of rumination play in the important academic challenge of learning after failure, as well as the potential cognitive and affective mechanisms underlying these effects.

Our conceptual approach was guided by Martin and Tesser’s Control Theory [8,9], which proposes that the rumination that arises in response to blocked goals can either hurt or help goal-oriented processing depending on whether attention is directed toward active, concrete means for problem solving (Reflection) or situated on one’s mood and problem history (Brooding). In support of the view that Reflection can be adaptive, we found that it positively influenced retest error correction. However, in contrast to this theory’s predictions, no negative (or positive) learning-related effects were found for the more passive style of Brooding. Nonetheless, despite the selective effects of Reflection on our measures of rebound from failure, Brooding and Reflection were both related to other key task variables, including first-test performance, subjective experiences in the task, and the late positive potential (LPP) to negative feedback—the ERP waveform we expected to be the most sensitive to rumination. For some of these measures (*i.e.*, first-test performance, FAEs, and the late LPP_neg_), Brooding and Reflection largely acted in expected, opposing ways to one another (or one acted in the expected direction and the other showed a null effect or trend in the opposite direction). Interestingly, for other measures (*i.e.*, RNTs and the early LPP_neg_), they exhibited more similar patterns.

With regard to the potential moderating influences of time on task and gender, we also found that these factors generally influenced the effects of Brooding and Reflection differently. For example, significant Brooding effects were only found when referencing later task blocks to the initial block (*i.e.*, change scores), suggesting that Brooding was most likely to emerge as a robust predictor after students had experienced persistent failure (due to the titration by the program) for a period of time. In contrast, with the exception of the effects on retest performance and the late LPP_neg_, the influence of Reflection occurred across the task as a whole, being equally evident during the initial blocks as during later ones. Finally, whereas the majority of Reflection-related effects did not differ as a function of gender, gender-specific effects were found for all relationships involving Brooding. In the discussion that follows, we address these findings in detail, highlighting the differential sensitivity of Reflection and Brooding to cognitive and affective measures, as well as to gender and time on task.

### 4.1. Reflection Benefits First-Test and Retest Memory

A tendency toward thinking about and purposefully analyzing the causes and outcomes of negative events (*i.e.*, Reflection) was associated with positive memory outcomes at both the first-test and retest. At first-test, trait Reflection predicted better performance for females, who as a group had performed at a lower level than males in their ability to generate correct answers to general knowledge questions. Indeed, females with higher levels of Reflection had levels of first-test performance within the range of males, whose performance did not appear to be influenced by rumination. Given that first-test performance was titrated by presenting the more difficult items from our question pool when students began performing above the target level (35%), these findings suggest that those females who reported higher trait Reflection were able to answer more difficult questions. Thus, it is possible that Reflection provided a protective or even facilitative effect for semantic retrieval in the face of persistent failure during the task. However, it is also possible that students with higher Reflection scores simply had higher levels of pre-existing semantic knowledge.

More direct support for the positive influence of Reflection on memory encoding and retrieval comes from our analysis of retest performance. These analyses, which controlled for performance at first-test, demonstrated beneficial effects of trait Reflection on rebound from failure in both males and females. Specifically, students who indicated they were more likely to Reflect in the context of negative situations were more likely to show improvement (or at least no decrement) in their ability to rebound from failure during the later blocks of the test compared to the initial block. For males, this manifested as higher Reflection scores mitigating the general tendency to show poorer error correction as the task progressed. For females, a trait tendency to Reflect was associated with greater improvements in error correction in the remainder of the task. Taken together, these results suggest that Reflective rumination allows individuals to maintain task-related effort in the face of challenge and failure, and is consistent with other research finding it to be associated with less distraction and interference by self-relevant stimuli [14].

Brooding was associated with marginally poorer first-test performance in females, but somewhat surprisingly, had no negative impact for either males or females on the ability to overwrite these incorrect answers and provide the correct responses at retest. This null effect for Brooding on learning the correct answer (and inhibiting retrieval of the incorrect answer) appears to contrast with previous studies where Brooding was found to impair the ability to update memories in the presence of emotional stimuli [48], or otherwise inhibit no-longer-useful material [59,60]. Although the failure to find a retest effect might be due to power issues stemming from our relatively small sample size, significant effects of Brooding did emerge elsewhere within this task, including effects on subjective experiences and ERP responses to errors. Thus, if an effect on error correction was masked by low power, then the effect size was most likely fairly small. Alternatively, presentation of the correct answer a few seconds after the negative feedback may actually have given brooding students an external stimulus that reoriented their attention, pulling them out of the negative mindset induced by the negative performance feedback.

Hertel and colleagues [61] found that brooders were equally as likely as non-brooders to later freely recall target words when a trigger for rumination was interrupted by a cue to interact with (*i.e.*, type) the word, bringing attention back to the target stimulus. However, brooders recalled fewer words when a cue to brood came after interaction with the word, perhaps because it disrupted focus on encoding. In the current task, presenting the correct answer after the negative feedback may have afforded brooders an explicit opportunity to redirect their attention externally toward corrective information. By this view, it is possible that if we had presented the correct answer simultaneously with the negative feedback (*i.e.*, as during the retest), brooders might have been more likely to focus attention on the negative outcome and directed attention internally to the feelings generated by it. If this internal focus came at the expense of externally-focused attention to the correct answer, such interference might have more likely resulted in Brooding-related impairments in rebound from failure.

If, for students with higher trait Reflection, corrective feedback may not have simply served as a distractor from negative thoughts, but rather, may have provided information directly relevant to their goal of purposefully thinking about and analyzing the causes of the error, we might expect Reflection-related modulation of the ERP response to the corrective feedback itself. However, no such relationship was found, at least when examining the portions of this waveform that demonstrated sensitivity to subsequent memory in our validation analysis (Supplemental S2.3; see also [19,20]). Although it is not clear why this relationship was lacking, it is possible that Reflection modulated encoding-relevant processes at sites or epochs other than the sites where memory-related processes were most evident in the sample as a whole. Additionally, it is important to consider that error correction on the retest is related to many factors that include not only how well items were initially encoded, but also post-encoding consolidation, as well as processing at the time of retest retrieval. Given that we measured rumination as a trait tendency, it is possible that such a habitual style of responding could have impacted these consolidation and/or retrieval processes, as well.

To our knowledge, only one other study has measured the influence of rumination on the use of feedback to improve performance in a similar type of sample [62]. In that study, following the presentation of false negative feedback after an attempt to complete a set of simple tasks (e.g., declaring creative uses for various objects, finding words in a word search task), the authors found that inducing action-focused rumination (e.g., instructing participants to think about how to their improve performance) *versus* state-focused rumination (e.g., instructing participants to think about how their poor skills on that task might impact their future) resulted in better performance on a second attempt at the task. State-focused rumination, if anything served to impair subsequent performance. To the extent that action- and state-focused rumination are similar to Reflection and Brooding, respectively, our findings echo their results, but also show how these types of effects may manifest as a function of trait rumination and in a task where learning is central.

### 4.2. Brooding and Reflection Influence Thoughts and Feelings in Response to Negative Feedback

To understand the effect of rumination on how students processed failure signals in this challenging task, we measured students’ response to negative feedback using both subjective self-reports and neural correlates previously associated with error detection (FRN; [22]) and motivated attention to arousing events (LPP; [63]). Reflection was associated with significant increases in recurring negative thoughts (RNTs), and Brooding with marginal increases. These effects could be viewed simply as a type of manipulation check showing that the task was effective in evoking ruminative tendencies in subjects more predisposed to ruminate. Notably, however, Reflection influenced this type of thinking throughout the task, for both genders, whereas the marginal effects of Brooding were only found when referencing later blocks to the first block (*i.e.*, change scores) and only in females. These different patterns might have arisen because our measure of RNTs focused only on the quantity of these thoughts rather than the specific content of these thoughts. In other words, the content of the thoughts might have been different for those high in Reflection and Brooding, consistent with the differences in analytical *versus* affective thinking characterizing these subtypes of rumination. Indeed, students’ feelings after errors (FAEs), a more affectively-focused measure, was only influenced by Brooding. For females, we found evidence of the expected pattern of relatively more negative FAEs for higher Brooding as the task progressed. Surprisingly, however, Brooding was associated with significantly less negative (more neutral) FAEs as the task progressed for males. As this is a self-report measure, it is not clear whether this finding indicates that males who were higher in Brooding were attempting to regulate negative feelings through affective disengagement or whether they might have been presenting a false front to mask actual distress from the persistent negative feedback of this task (see also [64]).

Turning to ERP measures, which presumably would be less influenced by demand characteristics, we found no effects of either rumination subtype in either gender on our earliest marker of error processing, the FRN. The lack of rumination effects on the FRN is consistent with our earlier prediction that rumination would modulate processes occurring after initial error detection, rather than those associated directly with the detection itself. Rumination is typically conceptualized as a reactive response to negative outcomes, rather than anticipatory vigilance for them, as would be more typical of worry [65,66]. Although null effects are always subject to criticisms regarding power and sensitivity, we note that this component was enhanced in males with higher pre-task depression (see Supplemental Section S1). This finding is consistent with past research finding the FRN to be sensitive to depressive mood [67,68], and lends support for the view that the FRN in the current study is otherwise representative of the typical FRN. Interestingly, we also found that the task-wide FRN_neg_ was enhanced in males who reported more neutral FAEs on average (see Supplemental Section S3), paralleling the paradoxical finding that Brooding was associated with increasingly more neutral FAEs. Taken together, these results suggest that the more salient the negative feedback, the more males were motivated to self-report that they were not negatively affected by this feedback.

With regard to the LPP_neg_, we found that for females, the early portion (400–600 ms) was similarly enhanced by both Reflection and Brooding, whereas the later portion (600–1000 ms) demonstrated a significant enhancement by Brooding in females, and a marginal attenuation by Reflection in both genders. Moreover, the significant effects of Reflection on the early LPP_neg_ were found across the task as a whole, whereas the effects of Brooding on both the early and late LPP_neg_ only emerged when examining change scores. Indeed, they were most pronounced in the block immediately following the initial block (*i.e.*, ΔB1-to-B2). Generally speaking, the emergence of rumination effects on the LPP, but not the FRN, corroborates behavioral findings showing that ruminators, and brooders in particular, tend to exhibit heightened arousal and protracted attention to negatively-valenced information (e.g., [12]). The LPP is thought to represent activity in extrastriate cortex that is amplified by feedback connections from the amygdala [69]. Correspondingly, past fMRI research has shown that ruminators demonstrate increased and prolonged neural activity in the amygdala, a subcortical, limbic brain structure responsible for emotional processing when viewing emotional stimuli [70,71].

The LPP has been shown to index not only spontaneous and passive (*i.e.*, bottom-up) responses to motivationally-relevant stimuli [72], but also more active and deliberate (*i.e.*, top-down) cognitive and affective appraisals [73]. Research on the time course of the LPP has shown that modulation by bottom-up perceptual processing of emotionally evocative stimuli can occur as early as 160 ms post-stimulus onset, whereas more active, top-down control over this emotional reaction (e.g., down-regulation through cognitive reappraisal) becomes apparent just after 600 ms [57]. Thus, it appears that females’ tendency towards Brooding facilitated a rapid “bottom-up” increase in arousal and attention to negative feedback (*i.e.*, early LPP), followed by further up-regulation of sustained attention and arousal (or at least prevention of the normal habituation to these signals) through top-down processes. Reflection, on the other hand, facilitated the rapid, “bottom-up” orienting response to negative outcomes, but unlike Brooding, it may have acted in a “top-down” fashion to down-regulate, or at least prevent further sustained arousal to this stimulus.

### 4.3. Differential Effects of Brooding and Reflection as a Function of Time on Task

Significant effects of Brooding emerged only after subjects had experienced failure in the initial block. Specifically, the significant impact of this ruminative style on both subjective self-reports and the LPP_neg_ was only found when referencing later blocks against the initial block (*i.e.*, change scores). In addition, whereas the effects of Brooding on feelings (FAEs) were found to be similar across all change scores as the task progressed, both the early and late LPP_neg_ appeared to show a particularly rapid reversal between the effects of Brooding in the initial block and the following block (Block 2). In contrast, significant effects of the Reflective style of rumination on first-task performance, RNTs and the early LPP_neg_ were apparent across the task as a whole. Only the influence of Reflection on error correction and the marginal impact on the late LPP_neg_ showed an effect that emerged through change scores.

To understand why these different patterns occurred it is useful to consider that we employed an “adaptive task” in which a titration algorithm created a persistent level of challenge and failure throughout the task, for all students, regardless of either their pre-existing knowledge or their in-task efforts. Students would have only become aware the constant difficulty of the task as they made their way through the first block, however, and found that whatever attempts they made to garner a higher percentage of correct responses were simply met with harder questions. Thus, from the perspective of the student, as the task progressed they not only may have been blocked in their general goal of performing well on the task, but also in improving their performance with increased effort. Brooding, which relates to a more passive fixation on the negative mood caused by a blocked goal, may have been particularly evident in the context of this additional goal discrepancy, when active strategies to improve performance failed. In contrast, for those with a tendency toward Reflection, even initial encounters with negative feedback triggered increases in RNTs and bottom-up attention toward negative feedback (*i.e.*, early LPP_neg_), leading these effects to emerge consistently across the task. However, when additional goal challenges compounded as the task progressed, the more action-focused ruminative style of those higher in Reflection may have been particularly useful for fostering more adaptive responses (*i.e.*, no greater up-regulation of the late LPP_neg_, enhanced error correction).

To our knowledge, this is the first study to demonstrate time-based effects of either rumination subtype within a single task. However, other studies indirectly support the view that time is a key factor in understanding the effects of rumination. First, rumination itself may persist over long periods, particularly when individuals feel far from attaining their goals, whether due to poor task performance or lack of task completion (e.g., [10,74]). Furthermore, according to Martin and Tesser [9], rumination and its influence on affect and cognition may become exacerbated over time depending on how one appraises goal blockage. For instance, viewing an inability to attain a short-term, lower-order goal (e.g., losing weight) as indicative of being unable to achieve a longer-term, higher-order goal (e.g., happiness, well-being) may actually interfere with the ability to attain both short-term and longer-term goals [75].

### 4.4. Differential Effects of Brooding and Reflection as a Function of Gender

It was notable that all Brooding-related effects showed gender differences, whereas gender effects were more mixed for Reflection. Specifically, for females only, Brooding resulted in less habituation of the early LPP_neg_ and greater up-regulation of the late LPP_neg_. It also marginally impaired females’ first-test performance and resulted in a trend toward increased RNTs and greater negative affect following errors. As discussed previously, the only significant effect of Brooding observed for males suggested that this subtype of rumination led to decreased, rather than increased negative feelings about errors—an effect in the opposite direction to that found for females. These female-specific effects of Brooding extend earlier clinically-focused work showing that dysphoric female ruminators are particularly vulnerable to depression and other negative consequences (e.g., perceived lower mastery over life events, increased chronic stress; [76]), as well as other empirical evidence that rumination tends to influence affect, cognition, and behavior primarily for females [15,77,78]. In contrast, the Reflection-related enhancements observed for error correction outcomes and RNTs were not gender-specific, suggesting that males and females were equally able to use the adaptive aspects of Reflective rumination in the service of encoding corrective information and overcoming failure.

Past studies have noted that females are more likely than males to feel less “in control” of their emotions and more likely to turn to rumination as a means of gaining back some control, even if in practice it does not necessarily serve as an adaptive means of emotion regulation [64]. In our present study with non-depressed females, however, there were no gender differences in pre-test Brooding or Reflection RRS subscores or the quantity of self-reported RNTs during the task. Thus, the lack of negative Brooding effects in males does not appear to stem simply from a quantitatively lower level of rumination. However, this does not rule out the possibility that the content and/or the manner of males’ rumination differed from that of female subjects. Some qualitative difference might underlie why Brooding enhanced the overall pattern of males’ growing indifference (*i.e.*, neutrality) to negative feedback (*i.e.*, FAEs), whereas for females, it maintained their pattern of continual concern and unpleasant feelings. Some studies show that males demonstrate greater challenges in recognizing, attending to, and interpreting negative emotional information compared to females [79,80]. Thus, scores on the Brooding scale may be less sensitive to measures in our task for males compared to females because the RRS asks respondents to merely consider *hypothetically* how they would ruminate over emotional events, and males can find these kinds of emotional judgments difficult [79].

## 5. Conclusions

Extending Martin and Tesser’s Control Theory [8,9], the findings from the current study support the view that the more deliberate and concrete thinking associated with the Reflection subtype of rumination serves to prevent sustained up-regulation of arousal to negative self-relevant information, and yields better outcomes for rebounding from failure (see also [6,11]). In contrast, the state and mood-focused Brooding subtype enhances females’ attention to signals indicating blocked goals (*i.e.*, negative feedback), particularly as these blockages persist and compound over time. Although Brooding did not have immediate effects on females’ (or males’) ability to remediate errors in the present study, it is possible that over time, desire to avoid this greater arousal to negative feedback would adversely affect the motivation to engage in challenging tasks where negative feedback might be anticipated. It would be worthwhile to extend both of these findings further by testing the longitudinal effects of rumination on learning and rebound from failure (e.g., over the course of an academic semester or school year; see also [81]), particularly in STEM fields where stereotype threat might exacerbate Brooding ruminative tendencies in females [29,30].

Even though these findings are largely aligned with patterns of rumination effects found elsewhere, they extend them in important ways. First, in addition to providing evidence for the general sensitivity of the LPP to rumination on failure signals (see also [29]), the high temporal resolution of ERPs afforded the ability to dissociate the effects of Brooding and Reflection on putative correlates of bottom-up and top-down attention. Specifically, although the bottom-up salience of negative feedback was enhanced for both subtypes of rumination, through top-down processes (*i.e.*, late LPP_neg_), Brooding further up-regulated females’ attention to this feedback, whereas Reflection showed some evidence of down-regulating this response. Second, we were able to demonstrate these and other meaningful cognitive and affective effects of rumination in an academically-relevant task and in non-depressed students. This supports the view that rumination not only has implications for clinical outcomes, but may contribute important non-cognitive variance to student outcomes, particularly when students experience persistent challenges to their goals of performing well.

## Figures and Tables

**Table 1:** Sample characteristics with mean scores of pre-test self-report questionnaires and demographics.

	Overall	Range	Males	Females
*n*	40	-	20	20
Age	20.43 (0.38)	18.16–29.91	20.87 (0.66)	19.99 (0.38)
Years of Education	14.20 (0.18)	13–16	14.20 (0.25)	14.20 (0.27)
BDI-II	7.45 (0.74)	0–17	7.80 (1.03)	7.10 (1.07)
RRS Total	39.23 (1.65)	23–69	38.80 (2.66)	39.65 (2.03)
Brooding	9.05 (0.46)	5–16	8.90 (0.76)	9.20 (0.52)
Reflection	9.28 (0.56)	5–19	9.50 (0.93)	9.05 (0.64)

Standard errors of the mean appear in parentheses here and in all subsequent tables.

**Table 2:** Memory Performance. Proportion correct at first-test and retest as a function of gender and block. Means are adjusted for BDI-II, Brooding, and Reflection covariates.

Behavior	Overall	Block 1	Block 2	Block 3	Block 4
First-Test Accuracy					
Females	0.340 (0.007)	0.346 (0.006)	0.349 (0.008)	0.335 (0.012)	0.328 (0.016)
Males	0.363 (0.007)	0.363 (0.006)	0.360 (0.008)	0.377 (0.012)	0.352 (0.016)
Retest Error Correction					
Females	0.606 (0.021)	0.578 (0.025)	0.643 (0.030)	0.605 (0.026)	0.598 (0.031)
Males	0.545 (0.021)	0.597 (0.025)	0.573 (0.030)	0.499 (0.026)	0.513 (0.031)

**Table 3:** Subjective experience. Means are adjusted for BDI-II, Brooding, and Reflection covariates.

Subjective Experience	Overall	Block 1	Block 2	Block 3	Block 4
Recurring Negative Thoughts					
Females	4.50 (0.40)	4.45 (0.40)	4.46 (0.42)	4.78 (0.51)	4.29 (0.51)
Males	3.97 (0.40)	4.55 (0.40)	4.09 (0.42)	3.62 (0.51)	3.61 (0.51)
Feelings After Errors					
Females	3.44 (0.24)	3.53 (0.27)	3.45 (0.35)	3.19 (0.29)	3.59 (0.31)
Males	3.66 (0.24)	2.98 (0.27)	3.48 (0.35)	3.96 (0.29)	4.21 (0.31)

Notes: Recurring Negative Thoughts were rated on a scale of 1 (none at all) to 9 (an extreme amount), with 5 representing a moderate amount. Feelings After Errors were rated on a scale of 1 (extremely unpleasant) to 9 (extremely pleasant), with 5 representing neither pleasant nor unpleasant.

**Table 4:** Event-Related Potential amplitudes (µV). Means are adjusted for BDI-II, Brooding, and Reflection covariates.

ERP	Overall	Block 1	Block 2	Block 3	Block 4
FRN_neg_					
Females	−2.18 (0.45)	−1.58 (0.53)	−1.83 (0.44)	−2.34 (0.54)	−2.97 (0.49)
Males	−2.53 (0.45)	−1.82 (0.53)	−2.48 (0.44)	−2.78 (0.54)	−3.06 (0.49)
FRN_diff_					
Females	−1.91 (0.33)	−1.18 (0.51)	−1.72 (0.47)	−2.38 (0.39)	−2.34 (0.42)
Males	−1.87 (0.33)	−1.71 (0.51)	−2.07 (0.47)	−1.83 (0.39)	−1.84 (0.42)
Early LPP_neg_					
Females	3.05 (0.29)	3.43 (0.32)	3.10 (0.31)	3.06 (0.34)	2.61 (0.31)
Males	1.96 (0.29)	2.43 (0.32)	2.02 (0.31)	1.70 (0.34)	1.68 (0.31)
Late LPP_neg_					
Females	2.09 (0.21)	1.98 (0.24)	2.26 (0.24)	2.09 (0.27)	2.02 (0.24)
Males	1.68 (0.21)	1.83 (0.24)	1.96 (0.24)	1.48 (0.27)	1.44 (0.24)
Post LERN					
Females	−3.22 (0.39)	−2.33 (0.34)	−3.47 (0.42)	−3.60 (0.43)	−3.47 (0.48)
Males	−2.75 (0.39)	−2.77 (0.34)	−2.94 (0.42)	−2.78 (0.43)	−2.51 (0.48)
Left LERN					
Females	−2.18 (0.28)	−1.50 (0.26)	−2.39 (0.30)	−2.32 (0.32)	−2.52 (0.33)
Males	−2.03 (0.28)	−1.82 (0.26)	−2.08 (0.30)	−2.22 (0.32)	−1.99 (0.33)

## Supplementary Materials

The following are available online at http://www.mdpi.com/2076-3425/6/1/7/s1, S1: Effects of Pre-Task Depression, S2: Validation of ERP Waveforms, S3: Correlations between Self-Reported Subjective Experiences and ERP Measures.

## References

[1] Bandura S.A. (1993). Perceived self-efficacy in cognitive development and functioning. Educ. Psychol..

[2] Duckworth A.L., Peterson C., Matthews M.D., Kelly D.R. (2007). Grit: Perseverence and passion for long-term goals. Personal. Process. Individ. Differ..

[3] Dolan R.J. (2002). Emotion, cognition, and behavior. Science.

[4] Pessoa L. (2010). Emergent processes in cognitive-emotional interactions. Dialogues Clin. Neurosci..

[5] Nolen-Hoeksema S., Morrow J. (1991). A prospective study of depression and posttraumatic stress symptoms after a natural disaster: The 1989 loma prieta earthquake. J. Personal. Soc. Psychol..

[6] Nolen-Hoeksema S., Wisco B.E., Lyubomirsky S. (2008). Rethinking rumination. Perspect. Psychol. Sci..

[7] Lyubomirsky S., Nolen-Hoeksema S. (1995). Effects of self-focused rumination on negative thinking and interpersonal problem solving. J. Personal. Soc. Psychol..

[8] Martin L., Tesser A., Uleman J.S., Bargh J.A. (1989). Toward a motivational and structural theory of ruminative thought. Unintened Thought.

[9] Martin L., Tesser A., Wyer R.S. (1996). Some ruminative thoughts. Ruminative Thoughts.

[10] Martin L.L. (1986). Set/reset: Use and disuse of concepts in impression formation. J. Personal. Soc. Psychol..

[11] Treynor W., Gonzalez R., Nolen-Hoeksema S. (2003). Rumination reconsidered: A psychometric analysis. Cogn. Ther. Res..

[12] Donaldson C., Lam D., Mathews A. (2007). Rumination and attention in major depression. Behav. Res. Ther..

[13] Vanderhasselt M.A., Kuhn S., de Raedt R. (2011). Healthy brooders employ more attentional resources when disengaging from the negative: An event-related fMRI study. Cogn. Affect. Behav. Neurosci..

[14] Daches S., Mor N., Winquist J., Gilboa-Schechtman E. (2010). Brooding and attentional control in processing self-relevant information: Evidence from a modified garner task. Cogn. Emot..

[15] Nolen-Hoeksema S. (1987). Sex differences in unipolar depression: Evidence and theory. Psychol. Bull..

[16] Nolen-Hoeksema S., Morrow J., Fredrickson B.L. (1993). Response styles and the duration of episodes of depressed mood. J. Abnorm. Psychol..

[17] Nolen-Hoeksema S., Parker L.E., Larson J. (1994). Ruminative coping with depressed mood following loss. J. Personal. Soc. Psychol..

[18] Sarin S., Abela J.R., Auerbach R.P. (2005). The response styles theory of depression: A test of specificity and causal mediation. Cogn. Emot..

[19] Butterfield B., Mangels J.A. (2003). Neural correlates of error detection and correction in a semantic retrieval task. Brain Res. Cogn. Brain Res..

[20] Mangels J.A., Butterfield B., Lamb J., Good C., Dweck C.S. (2006). Why do beliefs about intelligence influence learning success? A social cognitive neuroscience model. Soc. Cogn. Affect. Neurosci..

[21] Miltner W.H., Braun C.H., Coles M.G. (1997). Event-related brain potentials following incorrect feedback in a time-estimation task: Evidence for a “generic” neural system for error detection. J. Cogn. Neurosci..

[22] Simons R.F. (2010). The way of our errors: Theme and variations. Psychophysiology.

[23] Foti D., Hajcak G. (2008). Deconstructing reappraisal: Descriptions preceding arousing pictures modulate the subsequent neural response. J. Cogn. Neurosci..

[24] Schupp H., Cuthbert B., Bradley M., Hillman C., Hamm A., Lang P. (2004). Brain processes in emotional perception: Motivated attention. Cogn. Emot..

[25] Holroyd C.B., Coles M.G. (2002). The neural basis of human error processing: Reinforcement learning, dopamine, and the error-related negativity. Psychol. Rev..

[26] Moser J.S., Hajcak G., Bukay E., Simons R.F. (2006). Intentional modulation of emotional responding to unpleasant pictures: An ERP study. Psychophysiology.

[27] Hajcak G., Olvet D.M. (2008). The persistence of attention to emotion: Brain potentials during and after picture presentation. Emotion.

[28] Cuthbert B.N., Schupp H.T., Bradley M.M., Birbaumer N., Lang P.J. (2000). Brain potentials in affective picture processing: Covariation with autonomic arousal and affective report. Biol. Psychol..

[29] Mangels J.A., Good C., Whiteman R.C., Maniscalco B., Dweck C.S. (2012). Emotion blocks the path to learning under stereotype threat. Soc. Cogn. Affect. Neurosci..

[30] Beilock S.L., Rydell R.J., McConnell A.R. (2007). Stereotype threat and working memory: Mechanisms, alleviation, and spillover. J. Exp. Psychol. Gen..

[31] Schmader T., Johns M., Forbes C. (2008). An integrated process model of stereotype threat effects on performance. Psychol. Rev..

[32] Marco-Pallares J., Kramer U.M., Strehl S., Schroder A., Munte T.F. (2010). When decisions of others matter to me: An electrophysiological analysis. BMC Neurosci..

[33] Hertel P. (2004). Memory for emotional and nonemotional events in depression. Mem. Emot..

[34] Watkins E.R., Nolen-Hoeksema S. (2014). A habit-goal framework of depressive rumination. J. Abnorm. Psychol..

[35] Yang Q., Gu R., Tang P., Luo Y.J. (2013). How does cognitive reappraisal affect the response to gains and losses?. Psychophysiology.

[36] Binder J.R., Desai R.H., Graves W.W., Conant L.L. (2009). Where is the semantic system? A critical review and meta-analysis of 120 functional neuroimaging studies. Cereb. Cortex.

[37] Paller K.A., Wagner A.D. (2002). Observing the transformation of experience into memory. Trends Cogn. Sci..

[38] Kutas M., Federmeier K.D. (2000). Electrophysiology reveals semantic memory use in language comprehension. Trends Cogn. Sci..

[39] Nobre A.C., McCarthy G. (1995). Language-related field potentials in the anterior-medial temporal lobe: II. Effects of word type and semantic priming. J. Neurosci..

[40] Stern E.R., Mangels J.A. (2006). An electrophysiological investigation of preparatory attentional control in a spatial stroop task. J. Cogn. Neurosci..

[41] Johnson R., Nessler D., Friedman D. (2013). Temporally specific divided attention tasks in young adults reveal the temporal dynamics of episodic encoding failures in elderly adults. Psychol. Aging.

[42] Mangels J.A., Picton T.W., Craik F.I. (2001). Attention and successful episodic encoding: An event-related potential study. Brain Res. Cogn. Brain Res..

[43] Nessler D., Johnson R., Bersick M., Friedman D. (2006). On why the elderly have normal semantic retrieval but deficient episodic encoding: A study of left inferior frontal erp activity. NeuroImage.

[44] Beck A.T., Steer R.A., Brown G. (1996). Manual for Beck Depression Inventory II (bdi-ii).

[45] De Lissnyder E., Koster E.H., Goubert L., Onraedt T., Vanderhasselt M.A., de Raedt R. (2012). Cognitive control moderates the association between stress and rumination. J. Behav. Ther. Exp. Psychiatry.

[46] Zetsche U., Joormann J. (2011). Components of interference control predict depressive symptoms and rumination cross-sectionally and at six months follow-up. J. Behav. Ther. Exp. Psychiatry.

[47] Berman M.G., Peltier S., Nee D.E., Kross E., Deldin P.J., Jonides J. (2010). Depression, rumination and the default mode network. Soc. Cogn. Affect. Neurosci..

[48] Bernblum R., Mor N. (2010). Rumination and emotion-related biases in refreshing information. Emotion.

[49] Davis R.N., Nolen-Hoeksema S. (2000). Cognitive inflexibility among ruminators and nonruminators. Cogn. Ther. Res..

[50] Joormann J. (2006). Differential effects of rumination and dysphoria on the inhibition of irrelevant emotional material: Evidence from a negative priming task. Cogn. Ther. Res..

[51] Holm S. (1979). A simple sequentially rejective multiple test procedure. Scand. J. Stat..

[52] Olvet D.M., Hajcak G. (2009). The stability of error-related brain activity with increasing trials. Psychophysiology.

[53] San Martín R. (2012). Event-related potential studies of outcome processing and feedback-guided learning. Front. Hum. Neurosci..

[54] Holroyd C.B., Pakzad-Vaezi K.L., Krigolson O.E. (2008). The feedback correct-related positivity: Sensitivity of the event-related brain potential to unexpected positive feedback. Psychophysiology.

[55] Nieuwenhuis S., Slagter H.A., Geusau V., Alting N.J., Heslenfeld D.J., Holroyd C.B. (2005). Knowing good from bad: Differential activation of human cortical areas by positive and negative outcomes. Eur. J. Neurosci..

[56] Hajcak G., Moser J.S., Holroyd C.B., Simons R.F. (2006). The feedback-related negativity reflects the binary evaluation of good *versus* bad outcomes. Biol. Psychol..

[57] Hajcak G., Dunning J.P., Foti D. (2009). Motivated and controlled attention to emotion: Time-course of the late positive potential. Clin. Neurophysiol..

[58] Weinberg A., Hilgard J., Bartholow B.D., Hajcak G. (2012). Emotional targets: Evaluative categorization as a function of context and content. Int. J. Psychophysiol..

[59] De Lissnyder E., Koster E.H., Derakshan N., de Raedt R. (2010). The association between depressive symptoms and executive control impariments in response to emotional and non-emotional information. Cogn. Emot..

[60] De Lissnyder E., Derakshan N., de Raedt R., Koster E.H. (2011). Depressive symptoms and attentional control in a mixed antisaccade task: Specific effects of rumination. Cogn. Emot..

[61] Hertel P.T., Benbow A.A., Geraerts E. (2012). Brooding deficits in memory: Focusing attention improves subsequent recall. Cogn. Emot..

[62] Ciarocco N.J., Vohs K.D., Baumeister R.F. (2010). Some good news about rumination: Task-focused thinking after failure facilitates performance improvement. J. Soc. Clin. Psychol..

[63] Hajcak G., MacNamara A., Olvet D.M. (2010). Event-related potentials, emotion, and emotion regulation: An integrative review. Dev. Neuropsychol..

[64] Nolen-Hoeksema S., Jackson B. (2001). Mediators of the gender difference in rumination. Psychol. Women Q..

[65] Borkovec T.D., Hazlett-Stevens H., Diaz M.L. (1999). The role of positive beliefs about worry in generalized anxiety disorder and its treatment. Clin. Psychol. Psychother..

[66] McLaughlin K.A., Borkovec T.D., Sibrava N.J. (2007). The effects of worry and rumination on affect states and cognitive activity. Behav. Ther..

[67] Santesso D.L., Steele K.T., Bogdan R., Holmes A.J., Deveney C.M., Meites T.M., Pizzagalli D.A. (2008). Enhanced negative feedback responses in remitted depression. Neuroreport.

[68] Tucker D.M., Luu P., Frishkoff G., Quiring J., Poulsen C. (2003). Frontolimbic response to negative feedback in clinical depression. J. Abnorm. Psychol..

[69] Sabatinelli D., Lang P.J., Keil A., Bradley M.M. (2007). Emotional perception: Correlation of functional mri and event-related potentials. Cereb. Cortex.

[70] Siegle G.J., Steinhauer S.R., Thase M.E., Stenger V.A., Carter C.S. (2002). Can’t shake that feeling: Event-related fmri assessment of sustained amygdala activity in response to emotional information in depressed individuals. Biol. Psychiatry.

[71] Ray R.D., Ochsner K.N., Cooper J.C., Robertson E.R., Gabrieli J.D., Gross J.J. (2005). Individual differences in trait rumination and the neural systems supporting cognitive reappraisal. Cogn. Affect. Behav. Neurosci..

[72] Codispoti M., Ferrari V., Bradley M.M. (2007). Repetition and event-related potentials: Distinguishing early and late processes in affective picture perception. J. Cogn. Neurosci..

[73] Hajcak G., Moser J.S., Simons R.F. (2006). Attending to affect: Appraisal strategies modulate the electrocortical response to arousing pictures. Emotion.

[74] Lassiter G.D., Pezzo M.V., Apple K.J. (1993). The transmitter-persistence effect: A confounded discovery?. Psychol. Sci..

[75] McIntosh W.D., Martin L.L., Clark M.S. (1992). The cybernetics of happiness: The relation betewen goal attainment, rumination, and affect. Review of Personality and Social Psychology.

[76] Nolen-Hoeksema S., Larson J., Grayson C. (1999). Explaining the gender difference in depressive symptoms. J. Personal. Soc. Psychol..

[77] Johnson D.P., Whisman M.A. (2013). Gender differences in rumination: A meta-analysis. Personal. Individ. Differ..

[78] Nolen-Hoeksema S. (1990). Sex Differences in Depression.

[79] Thayer J., Johnsen B.H. (2000). Sex differences in judgement of facial affect: A multivariate analysis of recognition errors. Scand. J. Psychol..

[80] Thayer J.F., Rossy L.A., Ruiz-Padial E., Johnsen B.H. (2003). Gender differences in the relationship between emotional regulation and depressive symptoms. Cogn. Ther. Res..

[81] Sakamoto S., Kambara M., Tanno Y. (2001). Response styles and cognitive and affective symptoms of depression. Personal. Individ. Differ..

