# Supplementary material for: Rumination and Rebound from Failure as a Function of Gender and Time on Task
Source: Brain Sci. 2016 Feb 17;6(1):7. doi: 10.3390/brainsci6010007 (PMC4810177; doi:10.3390/brainsci6010007)
Supplement: Supplementary file 1 [file brainsci-06-00007-s001.docx]

Supplementary Materials: Rumination and Rebound from Failure as a Function of Gender and Time
on Task

Ronald C. Whiteman ^1,2,^* and Jennifer A. Mangels ^1,2^

S1. Effects of Pre-Task Depression

In the following sections, we describe any effects involving the control variable of depression (*i.e.*, Beck Depression Inventory II scores (BDI-II); [1]) that were found during our main analyses (those focusing on Brooding and Reflection RRS variables). To briefly summarize these effects, depression was not predictive of memory performance at either the first-test or the retest (all *F*s < 2.26, all *p*s > 0.11), nor did it predict the amplitude of the Learning Related Negativity (LERN; all *F*s < 2.03, all *p*s > 0.14). However, it did predict participants’ early ERP responses to performance feedback (FRN_diff_ and early LPP_neg_) and, to a lesser extent, subjects’ extended attention to this feedback (late LPP_neg_) and reports of recurrent negative thoughts. Interestingly, BDI-II did not appear to be related to feelings after errors (FAEs; all *F*s < 1.23, all *p*s > 0.27). We detail these findings further below.

S1.1. Recurring Negative Thoughts (RNTs)

There were no effects of depression on RNTs in any analyses involving block (all *F*s < 2.66,
all *p*s > 0.11). Indeed, the only effects that involved BDI-II scores, either as a main effect or interaction, occurred when we examined change scores, where we observed a marginal three-way interaction of depression, gender, and change, *F*(2, 64) = 2.90, *p* = 0.06, η*_p_*^2^ = 0.08. However, none of the parameter estimates for males or females at each change score survived Holm-Bonferroni corrections (all *p*s > 0.14).

S1.2. Feedback-Related Negativity (FRN)

BDI-II scores were not at all predictive of the FRN_neg_, whether using the block or change score analysis approach (all *F*s < 1.84, all *p*s > 0.18). When analyzing the FRN_diff_, we found a three-way interaction amongst block, gender and depression, *F*(3, 96) = 3.10, *p* < 0.05, η*_p_*^2^ = 0.09, but after applying *post hoc* Holm-Bonferroni corrections, none of the individual parameter estimates reached significance.


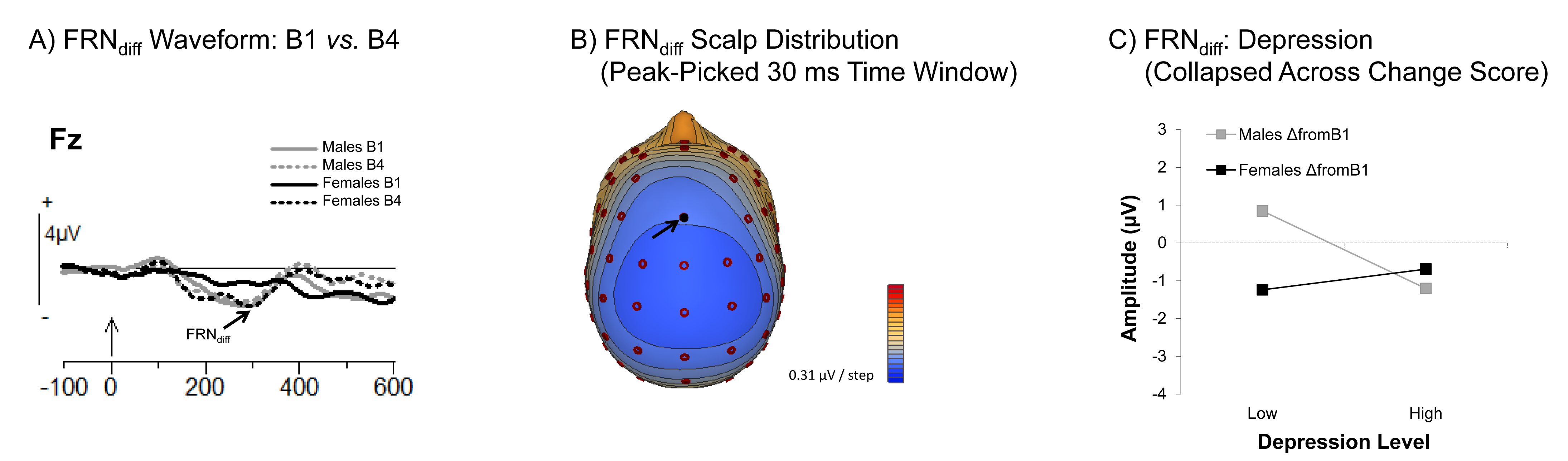


**Figure S1.** Feedback-Related Negativity difference wave (*i.e.*, FRN_diff_). (**A**) FRN_diff_ grand mean waveforms plotted at Fz for the first (B1) and last (B4) blocks, as a function of gender; (**B**) Scalp distribution of the FRN_diff_ for all subjects, collapsed over all four blocks. The arrow points to Fz, which is highlighted in black; (**C**) Effects of depression on change in FRN_diff_ amplitudes between Block 1 and subsequent blocks, as a function of gender.

Significance was found, however, when analyzing the FRN_diff_ with change scores (see
Figures S1A,B for a representation of the FRN_diff_ waveform and head map, respectively). With change scores, we found a significant gender by depression interaction, *F*(1, 32) = 4.64, *p* < 0.05, η*_p_*^2^ = 0.13, which *post hoc* analyses indicated was driven by a significant effect in males only, *β* = −0.51, *t* = 2.35, *p* < 0.05, η*_p_*^2^ = 0.15 (see Figure S1C). In other words, for males, increased pre-task levels of depression gave rise to a more negative-going FRN_diff_ in later blocks compared to Block 1. This finding was not present for females. The relationship between FRN_diff_ change scores and depression in males is consistent with findings elsewhere that mild-to-moderate levels of depression are associated with increased sensitivity to negative feedback signals, including failure (e.g., [2,3]).

S1.3. The Late Positive Potential (LPP)

S1.3.1. The Early LPP Response to Negative Feedback (Early LPP_neg_)

When investigating the effect of RRS scores on the early LPP_neg_ across all four blocks, we found a marginal two-way interaction between depression and gender, *F*(1, 32) = 2.97, *p* = 0.10, η*_p_*^2^ = 0.09. Somewhat surprisingly, *post hoc* exploration revealed that for females only, higher BDI-II scores predicted a decrease in early LPP_neg_ amplitudes across the task, *β* = −0.49, *t* = 2.72, *p* < 0.05, η*_p_*^2^ = 0.14, suggesting that arousal to negative feedback actually decreased as depression increased. When exploring the effects of RRS and BDI-II scores in the change score analysis, the effect of depression on the early LPP_neg_ differed for all participants as a function of change period, *F*(2, 64) = 3.15, *p* = 0.05, η*_p_*^2^ = 0.09. However, no single relationship of depression with the early LPP_neg_ within any given change period was significant after corrections for multiple comparisons (all *p*s > 0.26).

S1.3.2. The Late LPP Response to Negative Feedback (Late LPP_neg_)

BDI-II scores were not predictive of the late LPP_neg_ amplitude when analyzed as a function of block and gender (all *F*s < 2.12, all *p*s > 0.10). As we moved to the change score analysis approach, however, a marginal 2-way interaction between depression and change period emerged,
*F*(2, 64) = 2.61, *p* = 0.08, η*_p_*^2^ = 0.08, in addition to the effects found for Brooding and Reflection. Nonetheless, assessment of parameter estimates involving BDI-II scores revealed no significant coefficients within any given change period (all *p*s > 0.15).

S2. Validation of ERP Waveforms

S2.1. The Feedback-Related Negativity (FRN)

In order to validate that the FRN was differentially sensitive to errors, we compared the peak FRN measured during negative feedback (FRN_neg_) to the neural activity at the same electrode (Fz) and latency during positive feedback (FRN_pos_). Consistent with past research [4], the FRN_neg_ (*M* = −2.36, *SEM* = 0.31) was more negative-going than the FRN_pos_ (*M* = −0.47, *SEM* = 0.37). A 2 (feedback valence: positive *vs.* negative) × 2 (gender) × 4 (block) mixed-measures ANOVA confirmed a main effect of valence, *F*(1, 38) = 69.71, *p* < 0.001, η*_p_*^2^ = 0.65, that did not interact with either gender and/or block (all *F*s < 1.59, all *p*s > 0.19). However, a main effect of block, *F*(3, 114) = 6.70, *p* < 0.001, η*_p_*^2^ = 0.15, and a corresponding linear downward trend, *F*(1, 38) = 16.37, *p* < 0.001, η*_p_*^2^ = 0.30, revealed that both the FRN_neg_ and FRN_pos_ became more negative-going as the task progressed in both males and females. Thus, we also analyzed the difference between the FRN_neg_ and FRN_pos_ (*i.e.*, FRN_diff_), which provided a type of detrending of the FRN across the task. Indeed, when analyzing the FRN_diff_, the effect of block was eliminated; nor were there any effects of gender or any interactions (all *F*s < 1.51, all *p*s > 0.21).

S2.2. The Late Positive Potential (LPP)

S2.2.1. The Early LPP

The main goal of this analysis was to understand whether it was reasonable to collapse across electrodes in the posterior-superior region, thereby gaining greater stability in our LPP measurement than would be provided by a single electrode, without overcomplicating our primary analyses (*i.e.*, those including RRS) with the addition of region and hemisphere factors. Using electrode sites CP1/CPz/CP2 and P3/Pz/P4, we conducted a 3 (hemisphere: left, midline, right) × 2 (region: central-parietal, parietal) × 2 (feedback valence: negative *vs.* positive) × 2 (gender) × 4 (block) mixed-measures ANOVA on the amplitude of the early LPP (*i.e.*, from 400–600 ms) after the onset of performance feedback.

The spatial distribution of the early LPP was strongly right lateralized, as indicated by a main effect of hemisphere, *F*(1, 76) = 19.03, *p* < 0.001, η*_p_*^2^ = 0.33. This lateralization was particularly prominent over the more parietal electrodes, as indicated by *post hoc* analysis of a significant region by hemisphere interaction, *F*(2, 76) = 7.71, *p* < 0.005, η*_p_*^2^ = 0.17 (see also Figure 5b).

With regard to effects solely involving gender, block and/or valence, we found that females elicited a larger LPP than males, *F*(1, 38) = 7.19, *p* < 0.05, η*_p_*^2^ = 0.16 (Females: *M* = 3.41, *SEM* = 0.29; Males: *M* = 2.32, *SEM* = 0.29). In addition, regardless of gender, the early LPP was smaller for negative compared to positive feedback, *F*(1, 38) = 27.05, *p* < 0.001, η*_p_*^2^ = 0.42 (LPP_pos_: *M* = 3.23, *SEM* = 0.22; LPP_neg_: *M* = 2.50, *SEM* = 0.21), most likely because positive feedback was not only more rewarding, but also occurred less frequently (*i.e.*, ~35%; (see also [5]). Finally, the early LPP also differed as a function of block, *F*(2.51, 95.48) = 7.18, ε = 0.84, *p* < 0.001, η*_p_*^2^ = 0.16, an effect that was characterized by a downward linear trend, *F*(1, 38) = 17.67, *p* < 0.001, η*_p_*^2^ = 0.32, suggesting that this early index of sustained attention became attenuated as the task progressed. These three main effects were qualified, however, by a significant three-way interaction of gender, block and valence, *F*(3, 114) = 3.16, *p* < 0.05, η*_p_*^2^ = 0.08. *Post hoc* comparisons suggested that this interaction was driven by the finding that males showed the strongest block effects (*i.e.*, Block 1 *vs.* 4), and only for negative feedback.

Most importantly, however, we reviewed the results of the ANOVA for evidence of interactions between electrode site (*i.e.*, hemisphere and/or region) and our two experimental factors of interest (*i.e.*, gender and/or block). Such interactions would suggest that the distribution of the LPP differed across gender or time in the task, and thus, caution us against using the averaged LPP value in the 2 (gender) by 4 (block) mixed-measures Analysis of Covariance (ANCOVA) that included RRS and BDI-II as covariates. In contrast, main effects or interactions solely involving gender, block, and/or feedback valence, while interesting, would not preclude averaging across the six electrodes in our set, nor would variations in the amplitude of the LPP across regions or hemisphere that did not interact with gender and/or block. Finally, given that our main analyses would only include the LPP_neg_, if interactions did emerge that included valence alongside site, gender and/or block, we followed up these interactions by separately analyzing the LPP_neg_ and LPP_pos_, and only raised concerns about collapsing across sites if there were site x gender (or block) interactions in the LPP_neg_.

We found a significant three-way interaction amongst gender, valence and region, *F*(1, 38) = 4.40, *p* < 0.05, η*_p_*^2^ = 0.10, that superseded a significant valence by region interaction, *F*(1, 38) = 6.00, *p* < 0.05, η*_p_*^2^ = 0.14. When focusing on the LPP_neg_ alone, there was no longer a gender by region interaction, *F*(1, 38) = 0.13, *p* = 0.72, η*_p_*^2^ = 0.003, however. For the LPP_pos_, on the other hand, a marginal interaction effect emerged, *F*(1, 38) = 2.88, *p* = 0.10, η*_p_*^2^ = 0.07, whereby females’ amplitudes were significantly more positive (*M* = 3.85, *SEM* = 0.38) compared to males’ (*M* = 2.48, *SEM* = 0.25) in the anterior region (*i.e.*, central-parietal electrodes) only, *t*(38) = 2.97, *p* < 0.05. Feedback valence also appeared to marginally moderate regional distribution of activity across block, *F*(3, 114) = 2.23, *p* = 0.09, η*_p_*^2^ = 0.06. When separately examining the early LPP for positive and negative feedback, however, amplitudes within each region did not differ across blocks for either valence (Negative feedback: *F*(3, 114) = 1.57, *p* = 0.20, η*_p_*^2^ = 0.04; Positive feedback: *F*(2.59, 98.49) = 1.64, ε = 0.86, *p* = 0.19, η*_p_*^2^ = 0.04).

Taken together, these results suggest that the electrode sites specified in this 2 (region) by
3 (hemisphere) montage effectively captured early LPP amplitudes to negative feedback that remained consistent within every block and did not differ for either males or females, whether overall or by block. Thus, this analysis validates use of this averaged six-electrode cluster as a measure of the early LPP_neg_ in our investigation of brooding and reflection.

S2.2.2. The Late LPP

For the late LPP, we took an analysis approach similar to that described in S2.2.1. Similar to the early LPP, the late LPP was larger for positive feedback than for negative feedback, *F*(1, 38) = 11.12,
*p* < 0.005, η*_p_*^2^ = 0.23 (LPP_pos_: *M* = 2.24, *SEM* = 0.15; LPP_neg_: *M* = 1.88, *SEM* = 0.15), and differed across blocks regardless of feedback valence, *F*(3, 114) = 3.51, *p* < 0.05, η*_p_*^2^ = 0.09. This block effect, again, was described by a significant downward linear trend in amplitude across the task, *F*(1, 38) = 5.53, *p* < 0.05, η*_p_*^2^ = 0.13. In contrast with the early LPP, however, the late LPP did not differ significantly for males and females, *F*(1, 38) = 2.69, *p* = 0.11, η*_p_*^2^ = 0.07. Additionally, the spatial distribution of the late LPP differed somewhat in that it was generally largest along the midline, as indicated by *post hoc* analyses of a significant hemisphere effect, *F*(2, 76) = 18.67, *p* < 0.001, η*_p_*^2^ = 0.33, and larger over central-parietal compared to parietal electrodes, *F*(1, 38) = 8.23, *p* < 0.01, η*_p_*^2^ = 0.18. In addition, as can be seen in Figure 5b, the enhanced amplitude for central-parietal electrodes was most prominent at the lateral sites (*i.e.*, CPz and Pz did not differ), as supported by a *post hoc* comparisons of a significant region by hemisphere interaction, *F*(2, 76) = 12.09, *p* < 0.001, η*_p_*^2^ = 0.24. Finally, although there was a marginal valence, gender, and block interaction, *F*(2.53, 95.96) = 2.49, ε = 0.84, *p* = 0.08, η*_p_*^2^ = 0.06, after parsing all possible comparisons and correcting for multiple comparisons there were no significant effects.

With regard to our main concern of whether it was permissible to collapse across these
six electrodes, we found no three-, four- or five-way interaction effects involving any of the experimental variables and electrode site variables (all *F*s < 0.88, all *p*s > 0.35). These null effects support the view that it was valid to collapse across region and hemisphere in order to create an averaged late LPP for our primary analyses.

S2.3. “Difference Due to Memory” (Dm) ERP Effects

We conducted a traditional “difference due to memory (Dm)” analysis [6] in order to validate the electrode sites and epoch that differentiated all later corrected from all later not corrected items across the experiment as a whole. Specifically, ERPs time-locked to the initial presentation of the learning-relevant feedback (*i.e.*, correct answer) on error trials were back-sorted based on whether each answer was later retrieved successfully (or not) when the relevant question was re-probed on the subsequent surprise retest.

Consistent with where subsequent memory effects are typically found in this paradigm [7,8], our analysis focused on inferior temporal sites along the anterior (F7/8, FT9/10, T7/8) and posterior (TP9/10, CB1/2, O1/2) regions bilaterally from 500–1000 ms post-stimulus. Mean amplitude values were entered into a 2 (memory: later recalled/corrected, later not recalled/corrected) × 2 (region: anterior, posterior) × 2 (hemisphere: left, right) × 3 (electrode) × 2 (gender) mixed-measures ANOVA. A significant main effect of memory was found, *F*(1, 38) = 41.89, *p* < .001, η*_p_*^2^ = 0.52, whereby items subsequently remembered elicited a more negative-going ERP at all electrode sites compared to items subsequently forgotten. However, this main effect was qualified by a two-way interaction of memory with region, *F*(1, 38) = 11.54, *p* < .005, η*_p_*^2^ = 0.23, and a two-way interaction of memory with hemisphere, *F*(1, 38) = 4.76, *p* < .05, η*_p_*^2^ = 0.11. The 3-way interaction of memory, region and hemisphere did not reach significance, *F*(1, 38) = 2.69, *p* = 0.11, η*_p_*^2^ = 0.07. *Post hoc* exploration of the two-way interactions localized Dm effects to the posterior region (TP9/TP10, CB1/CB2, and O1/O2), and the left hemisphere (F7, FT9, T7, TP9, CB1, O1), respectively. Importantly, neither of these two two-way interactions varied as a function of gender and/or electrode (all *F*s < 0.87, all *p*s > 0.37), thus allowing us to justify collapsing over each 6-electrode cluster to create a more robust measure of both the posterior region and left hemisphere Dm effects when investigating the effects of Brooding and Reflection on learning-related feedback activity. Figure S2A illustrates the overall Dm effect at electrode CB1, plotted as a function of gender. Figure S2B shows the scalp topography for this effect, collapsed across block and gender, and averaged across the 500–1000 ms period following the onset of learning-relevant feedback.


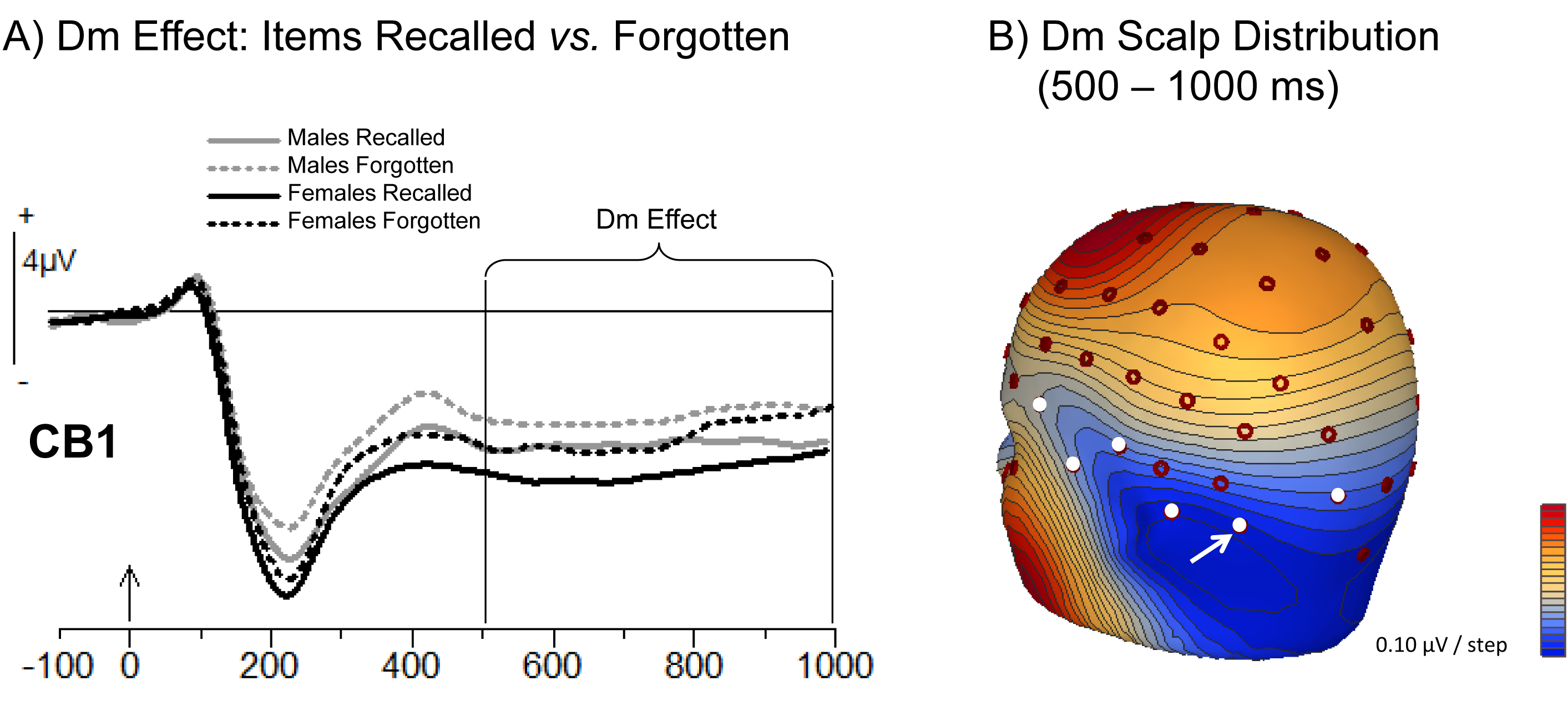


**Figure S2.** Difference due to memory (Dm). (**A**) Dm grand mean waveforms plotted at CB1 for the first (B1) and last (B4) blocks, as a function of gender; (**B**) Scalp distribution of the Dm effect plotted for all subjects, collapsed across all four blocks. Left hemisphere electrodes included in the analysis are highlighted in white, with an arrow pointing to CB1.

S3. Correlations between Self-Reported Subjective Experiences and ERP Measures

After z-scoring all variables, we conducted a series of zero-order correlations to explore potential relationships between subjects’ self-reported subjective experiences, their ERP responses to feedback (FRN, LPP, LERN), and behavioral performance on the retest. Given the gender-specific effects in our primary analyses, we split the group into males and females before conducting these analyses. However, for the sake of simplicity, correlations were only examined at the task (overall) level, rather than by block or using change scores.

No significant relationships between subjective experiences and either the early or late LPP_neg_ were found for either males or females (all *p*s > 0.15). However, both FAEs and RNTs were related to the FRN and the LERN, at least in male participants.

Surprisingly, for males, a more negative-going FRN to negative feedback (FRN_neg_) was associated with more neutral (less negative) self-reported affect following errors (FAEs), *r* = −0.57,
*p* < 0.01. Similarly, a more negative-going FRN_diff_ was marginally related to fewer self-reported RNTs, *r* = 0.41, *p* = 0.08. However, males with more RNTs (but not FAEs) exhibited more positive-going (*i.e.*, attenuated) LERN waveforms (Posterior LERN: *r* = 0.60, *p* < 0.01; Left LERN: *r* = 0.64, *p* < 0.005), suggesting they engaged less with the learning opportunity presented in the task (although no correlations were found with retest error correction, all *p*s > 0.40). For females, we found only a single significant relationship between their subjective experiences and feedback-related ERPs; females who reported more RNTs during the task also elicited a more negative-going FRN_neg_, *r* = −0.48, *p* < 0.05. Neither RNTs nor FAEs were related to LERN amplitudes or error correction rates in this group (all *p*s > 0.56).

Taken together, the results corroborate other findings indicating that females who focused more on negative thoughts during the task also expressed enhanced sensitivity to negative feedback. For males, however, self-reports and FRN measures appeared to exhibit an indirect (opposing) relationship, although a more predictable relationship between RNTs and the LERN was found. These gender differences may help explain some of the gender differences in rumination effects that were found (refer to Subsection 4.2).

References

1. Beck, A.T.; Steer, R.A.; Brown, G.K. *Manual for the Beck Depression Inventory*; 2 ed.; The Psychological Association: San Antonio, TX, USA, 1996.
2. Santesso, D.L.; Steele, K.T.; Bogdan, R.; Holmes, A.J.; Deveney, C.M.; Meites, T.M.; Pizzagalli, D.A. Enhanced negative feedback responses in remitted depression. *Neuroreport* **2008**, *19*, 1045–1048.
3. Tucker, D.M.; Luu, P.; Frishkoff, G.; Quiring, J.; Poulsen, C. Frontolimbic response to negative feedback in clinical depression. *J. Abnorm. Psychol.* **2003**, *112*, 667–678.
4. Hajcak, G.; Moser, J.S.; Holroyd, C.B.; Simons, R.F. The feedback-related negativity reflects the binary evaluation of good *versus* bad outcomes. *Biol. Psychol.* **2006**, *71*, 148–154.
5. Ito, T.A.; Thompson, E.; Cacioppo, J.T. Tracking the timecourse of social perception: The effects of racial cues on event-related brain potentials. *Personal. Soc. Psychol. Bull.* **2004**, *30*, 1267–1280.
6. Paller, K.A.; Wagner, A.D. Observing the transformation of experience into memory. *Trends Cogn. Sci.* **2002**, *6*, 93–102.
7. Butterfield, B.; Mangels, J.A. Neural correlates of error detection and correction in a semantic retrieval task. *Brain Res. Cogn. Brain Res.* **2003**, *17*, 793–817.
8. Mangels, J.A.; Butterfield, B.; Lamb, J.; Good, C.; Dweck, C.S. Why do beliefs about intelligence influence learning success? A social cognitive neuroscience model. *Soc. Cogn. Affect. Neurosci.* **2006**, *1*, 75–86.

© 2016 by the authors; licensee MDPI, Basel, Switzerland. This article is an open access article distributed under the terms and conditions of the Creative Commons by Attribution (CC-BY) license (http://creativecommons.org/licenses/by/4.0/).
